# Genome of the webworm *Hyphantria cunea* unveils genetic adaptations supporting its rapid invasion and spread

**DOI:** 10.1186/s12864-020-6629-6

**Published:** 2020-03-18

**Authors:** Qi Chen, Hanbo Zhao, Ming Wen, Jiaxin Li, Haifeng Zhou, Jiatong Wang, Yuxin Zhou, Yulin Liu, Lixin Du, Hui Kang, Jian Zhang, Rui Cao, Xiaoming Xu, Jing-Jiang Zhou, Bingzhong Ren, Yinliang Wang

**Affiliations:** 1 Jilin Provincial Key Laboratory of Animal Resource Conservation and Utilization, Northeast Normal University, Changchun, Jilin China; 2 Key Laboratory of Vegetation Ecology, MOE, Northeast Normal University, Changchun, China; 3 School of Life Sciences, Changchun Normal University, Changchun, Jilin China; 4 Meihekou Forest Pest Control Station, Changchun, Jilin China; 5 Garden and Plant Protection Station of Changchun, Changchun, Jilin China; 6 Rothamsted Research, Harpenden, AL5 2JQ UK

**Keywords:** Genome, Metagenome, Genetics, Fall webworm, Molecular evolution, Adaptation, Gene expansion

## Abstract

**Background:**

The fall webworm *Hyphantria cunea* is an invasive and polyphagous defoliator pest that feeds on nearly any type of deciduous tree worldwide. The silk web of *H. cunea* aids its aggregating behavior, provides thermal regulation and is regarded as one of causes for its rapid spread. In addition, both chemosensory and detoxification genes are vital for host adaptation in insects.

**Results:**

Here, a high-quality genome of *H. cunea* was obtained. Silk-web-related genes were identified from the genome, and successful silencing of the silk protein gene *HcunFib-H* resulted in a significant decrease in silk web shelter production. The CAFE analysis showed that some chemosensory and detoxification gene families, such as *CSPs*, *CCEs*, *GSTs* and *UGTs*, were expanded. A transcriptome analysis using the newly sequenced *H. cunea* genome showed that most chemosensory genes were specifically expressed in the antennae, while most detoxification genes were highly expressed during the feeding peak. Moreover, we found that many nutrient-related genes and one detoxification gene, *HcunP450* (CYP306A1), were under significant positive selection, suggesting a crucial role of these genes in host adaptation in *H. cunea*. At the metagenomic level, several microbial communities in *H. cunea* gut and their metabolic pathways might be beneficial to *H. cunea* for nutrient metabolism and detoxification, and might also contribute to its host adaptation.

**Conclusions:**

These findings explain the host and environmental adaptations of *H. cunea* at the genetic level and provide partial evidence for the cause of its rapid invasion and potential gene targets for innovative pest management strategies.

## Background

The fall webworm, *Hyphantria cunea* Drury (Erebidae: Hyphantria), is a polyphagous pest species in forest and agricultural ecosystems; where its larvae feed on most deciduous tree leaves [1]. When trees are infested, the fall webworm consumes nearly all leaves and causes great ecological and economic impact to the forest industry [2]. *H. cunea* is also an invasive pest, native to North America, but has spread globally in the past seven decades [3]. Behavioural, physiological and ecological adaptations present in this species are believed to contribute to its rapid spread.

First, the fall webworm has an extremely wide range of host plants and been reported to forage on more than 600 plant species, covering nearly all types of deciduous trees, especially mulberry, boxelder, walnut, sycamore, apple, plum, cherry, and elm [4].. Insect host selection is regulated by the chemosensory systems [5], especially for polyphagous herbivores [6–8]. Insect chemosensory systems consist of several gene families, including odorant receptor (*OR*), gustatory receptor (*GR*), ionotropic receptor (*IR*), chemosensory protein (*CSP*) and odorant binding protein (*OBP*) families. These genes encode proteins that participate in host plant detection and sexual communication [9–12]. Previous investigations have suggested that the large expansions in chemosensory gene families are a possible adaptation mechanism which enables polyphagy in the lepidopteran insect *Spodoptera frugiperda* [13] and other taxa such as *Apis mellifera*, *Bombyx mori* and *Bemisia tabaci* [9, 14–18]. Thus, chemosensory genes were further examined in this study to explore the roles of these genes in host plant adaptation of *H. cunea*. In addition, several studies have shown that the host ranges of insects are determined by their detoxification abilities [19, 20], which also contribute to adaptation to polyphagy in insect herbivores [13, 21]. Therefore, detoxification genes such as UDP- glycosyltransferase (*UGT*), glutathione S-transferase (*GST*), carboxyl/choline esterases (*CCE*), ATP-binding cassette transporter (*ABC*) and cytochrome P450 (*P450*) were screened from the transcriptome and metagenome datasets of *H. cunea* and analyzed for differential expression and positive selection.

Second, the fall webworm has a high reproductive capacity and a strong tolerance of extreme environments, including a wide range of temperatures (− 16 °C to 40 °C) and starvation (the larvae of fall webworm can live without food for more than 10 days) [22]. Numerous studies have found that the gut bacteria of insects play crucial roles in environmental adaptation by their insect hosts [23–25]. Gut microbes with a mutualistic relationship to their hosts contribute to preventing pathogen growth in insects [26]. For example, the gut bacteria of the desert locust *Schistocerca gregaria* could protect the locust gut from colonization by an insect pathogenic bacterium, *Serratia marcescens* [27]. Furthermore, gut microbial partnerships could help their insect hosts proliferate under a range of temperatures [28], conferring cold tolerance [29] and heat stress tolerance [30, 31]. Meanwhile, some gut bacteria and the natural products extracted from bacteria are used for pest control [23, 32]. Therefore, to gain new insights into the environmental adaptations of the fall webworm at the microbiome level, the compositional diversity of the gut microbiota in *H. cunea* was also investigated by metagenomic analysis in this study.

Finally, *H. cunea* larvae aggregate by creating silk webs on tree branches, this social behavior provides temperature regulation and protects them from predators [33, 34]. In most Lepidopteran species, the silk is composed of two major silk proteins, fibroin and sericin [35–37]. The fibroins form filaments, and the sericins seal and glue the filaments into fibers [37]. In caddisflies, the phosphorylation of fibroins was found to contribute to larval adaptation to aquatic habitats, suggesting that fibroin might be involved in environmental adaptation among silk-spinning insects [38]. Thus, we annotated in the *H. cunea* genome and identified genes from the silk gland, especially the silk proteins (fibroins and sericins) to explore the functions of these genes in *H. cunea*.

With the explosive growth of bioinformatics and sequencing technologies, many insect genomes have been sequenced and provided comprehensive information on the phylogeny, evolution, population geography, gene function and genetic adaptation of these insects. In Lepidoptera, at least ten species’ genomes have been sequenced and published [39–46]. Wu et al. had performed a genome study on *Hyphantria cunea* and provided some insights into the rapid adaptation of the fall webworm to changing environments and host plants [47], in this study, a higher quality genome sequence of *H. cunea* was obtained by using a mix of PacBio and Illumina platform. Moreover, some evidences suggested that the gut bacteria of insects played essential roles in the adaptation of insects to their host plant [23–25], thus a further metagenomic analysis was performed in *H. cunea*, the results might provided us a better understanding of its rapid spread and also some potential gene targets for developing new methods to manage this worldwide pest.

## Results

### Overview of genome assembly and annotation

The genome survey with k-mer analysis (Figure S1) showed that there is a small peak in depth = 22 which represented the heterozygous sequences, while the average k-mer depth was 45, and the peak indepth = 90 indicates the repetitive sequences. As a results, the tentative genome size of *H. cunea* was 563.96 Mb with a low heterozygosity of 0.23% and repetitive elements of 36.20% of the whole genome (Figure S1 and Table S1).

**Fig. 1:**
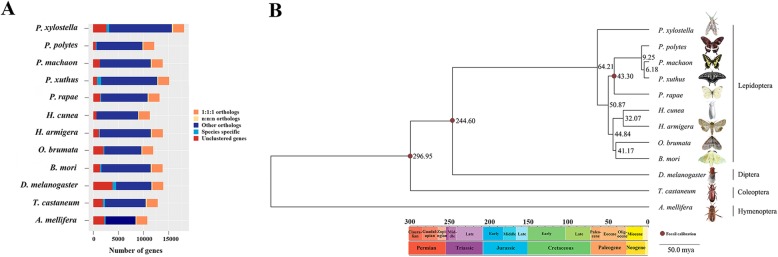
Overview of the *H. cunea* genome. **a** Types and numbers of homologous gene families among twelve species. **b** Maximum likelihood phylogenetic analysis among twelve insect species based on genomic data. The twelve species are *Apis mellifera*, *Bombyx mori*, *Drosophila melanogaster*, *Helicoverpa armigera*, *Hyphantria cunea*, *Operophtera brumata*, *Papilio machaon*, *Papilio polytes*, *Papilio xuthus*, *Pieris rapae*, *Tribolium castaneum* and *Plutella xylostella*. The numbers next to the nodes are the estimated node ages in million year (scale is 50.0 million years), and the colored box below indicates the geochronologic scale from Permian to Neogene

The generated genome assembly of *H. cunea* comprises a 559.30 Mb sequence with a 3.09 Mb contig N50. It contains 198.97 Mb of repetitive elements that occupy 35.71% of the genome. After correction with RNA sequencing data from 12 samples of different tissues and stages of *H. cunea*, we obtained 15,319 genes using three gene prediction strategies (Figure S2), 94.42% of which could be annotated and enriched by the GO and KOG databases (Figures S3 and S4), and the distribution of Nr homologous genes with the *H. cunea* genome in insect species was showed in Figure S5. Moreover, 637 tRNAs, 71 rRNAs, 48 miRNAs and 300 pseudogenes were predicted from the Rfam and miRBase databases by the Infernal, tRNAscan-SE and GenBlastA software (Table S2). Further analyses showed that 94.54 and 92.96% eukaryotic conserved genes were found in the genome of *H. cunea* by CEGMA and BUSCO, respectively, suggesting that the genome sequence we obtained was largely complete (Tables S3-S6). The genome of *H. cunea* possesses a comparatively longer contig N50 among all genomes of Lepidoptera species sequenced so far, the top 4 are as follows: *Operophtera brumata* (6.38 Mb) [40], *Spodoptera frugiperda* (5.6 Mb) [48], *Papilio bianor* (5.5 Mb) [49], and *H. cunea* (3.09 Mb), further confirming the high quality of the genome sequence of *H. cunea* (Table 1). Homology analysis of the *H. cunea* genome led to the identification of 2142 pairs of one-to-one single-copy orthologs among twelve species. This ortholog dataset was used for further studies described below. Only 27 genes were specific to *H. cunea*, which is the smallest species-specific number among the eight lepidopteran species (Fig. 1a).

**Table 1:** Overview of sequenced lepidopteran genomes

Species	Assembly size (MB)	Protein-coding Gene number	Contig N50 (MB)	GC (%)	Intron (%)	Repeat (%)	Protein number	Pseudogenes
*Plutella xylostella*	393.47	19,340	1.85	39.8	30.70	34	21,661	145
*Papilio polytes*	227.02	13,301	4.78	34	24.8	n.a.	16,620	66
*Papilio machaon*	278.42	14,850	0.08	34.4	n.a.	n.a.	17,745	102
*Papilio xuthus*	243.23	15,322	0.49	34.9	45.5	n.a.	21,602	232
*Pieris rapae*	245.87	13,152	1.42	33	33.3	22.7	18,966	70
*Hyphantria cunea*	559.30	15,319	3.09	36.57	29.1	35.71	18,207	300
*Helicoverpa armigera*	337.07	15,081	2.35	37.5	39.3	14.6	21,035	65
*Operophtera brumata*	638.21	16,912	2.88	37.8	17.7	53.5	16,912	n.a.
*Bombyx mori*	481.82	16,166	1.55	38.8	16.3	43.6	22,571	64

### Phylogeny of Lepidoptera

RAxML was used to construct a maximum likelihood phylogenetic tree using the 2142 single-copy orthologs among twelve insects whose genome sequences were available; eight Lepidoptera were included, while Hymenoptera (*A. mellifera*), Coleoptera (*T. castaneum*) and Diptera (*D. melanogaster*) were used as outgroups. The results showed that all nodes were supported by strong bootstrap values of 100%, and the topology of the higher taxa was consistent with those of previous phylogenetic studies [50, 51]. The results revealed that Lepidoptera was closer to Diptera, while Hymenoptera was located at the basal branch and formed a single clade (Fig. 1b). Within Lepidoptera, Papilionoidea (butterflies) formed a single clade, and *P. xylostella* (Yponomeutoidea) was separated from other moth taxa (Noctuoidea, Geometroidea and Bombycoidea). *H. cunea* was shown to be most closely related to *H. armigera*, which also belongs to the superfamily Noctuoidea. These results are in agreement with those obtained from the phylogenetic studies of Lepidoptera based on morphology and molecular data [52, 53]. The phylogenetic analysis indicated that Lepidoptera diverged from Diptera approximately 244.60 million years ago, which is consistent with the previously reported divergence time [50]. In Lepidoptera, the divergent time between the moths and butterflies in our study and was at Paleogene period, which is consist with Kawahara’s work, moreover, the genetic relationship between GEOMETROIDEA and BOMBYCOIDEA were close related, and they were grouped together with NOCTUOIDEA as well [54]. *H. cunea* and *H. armigera* were estimated to have diverged at the Eocene-Oligocene boundary with a divergence time of approximately 32.07 million years ago. The period from the late Eocene to early Oligocene has been considered as an important transition time and a link between the archaic world of the tropical Eocene and the more modern ecosystems of the Miocene [55].

### Expansion of chemosensory and detoxification gene families

To further explore host adaptation, the *H. cunea* gene families related to chemosensory abilities (*ORs*, *GRs*, *IRs*, *CSPs* and *OBPs*) were studied. With the combination of de novo assembly, homology-based search and RNA sequencing annotation, 72 *ORs*, 46 *GRs*, 66 *OBPs*, 20 *CSPs* and 21 *IRs* were identified in the *H. cunea* genome (Table 2). This result increased the number of chemosensory genes in *H. cunea* from the previous identifications via antennal transcriptome studies, which reported 52 *ORs*, 9 *GRs*, 30 *OBPs*, 17 *CSPs* and 14 *IRs* [56]. For the gene families related to detoxification, 32 *UGTs*, 25 *GSTs*, 75 *CCEs*, 95 *ABCs* and 109 *P450s* were identified using the same strategy as above (Table 2). The numbers of chemosensory and detoxification genes in *H. cunea* were further compared with those of some lepidopteran insects (Table 2) [46].

**Table 2:** The number of chemosensory and detoxification genes of *H. cunea* and other insect

*Gene name*	*Hyphantria. cunea*	*Papilio machaon*	*Operophtera brumata*	*Helicoverpa armigera*	*Bombyx mori*
CSPs	20	23	13	22	21
OBPs	66	29	15	30	43
ORs	72	51	29	74	73
GRs	46	10	9	19	76
IRs	21	33	31	52	31
P450s	109	112	133	112	83
GSTs	25	11	11	11	26
CCEs	74	53	42	53	73
APNs	24	17	28	17	14
ABCs	78	120	90	124	51
UGTs	32	39	11	39	45

Gene family expansion/contraction analyses showed that the *CSP*, *CCE*, *GST* and *UGT* gene families were expanded in *H. cunea* compared to the tested Lepidopteran species, as the divergence sizes were all significantly lower than the species sizes for these genes (Table 3). *CSPs* contribute to transportation, sensitivity and possibly the selectivity of the insect olfactory system [10]. In our study, an expansion of *CSPs* was detected, suggesting that they might relate to host plant selection of *H. cunea*, but much more testing is required. Among the detoxification gene families, *UGT*, *CCE* and *GST* families were found to be expanded in *H. cunea* (Table 3). Some studies also found that in some polyphagous species in Noctuoidea *GST*s and *CCE*s were greatly expanded, such as *H. zea*, *H. armigera* and *S. litura* [45, 46].

**Table 3:** Gene families expanded in *H. cunea* as calculated by CAFE

Divergence size	Species size	***P***-value	Gene ID	Annotation
2	4	0.006	EVM0007968 EVM0014260 EVM0001423.	Adenosine deaminase-related growth factor A
2	4	<1e-7	EVM0003438 EVM0001582 EVM0008984 EVM0011535	ATP-dependent helicase YHR031C
2	3	0.002	EVM0003192 EVM0011563 EVM0002688	Cytoskeleton
1	2	<1e-7	EVM0000776	Kazal-type serine proteinase inhibitor 1; gag-like protein
2	3	0.001	EVM0015065 EVM0001703 EVM0008164	Uncharacterized protein
1	2	<1e-7	EVM0000473 EVM0004248	Retroelement polyprotein
2	3	0.021	EVM0008576 EVM0007758 EVM0001530	Carboxyl/choline esterase CCE033a
1	2	0.047	EVM0012638 EVM0014428	PREDICTED: serine/threonine-protein kinase SMG1-like
1	2	0.041	EVM0011073 EVM0014934	Uncharacterized protein
1	2	<1e-7	EVM0014320 EVM0009912	Uncharacterized protein LOC103572275
1	2	0.021	EVM0001502 EVM0011366	Hypothetical protein KGM_05165
1	2	<1e-7	EVM0009792 EVM0013551	Uncharacterized protein LOC101742343
1	3	<1e-7	EVM0002515 EVM0008850	Uncharacterized protein
1	2	0.021	EVM0014968 EVM0000652	Lysosomal-trafficking regulator-like
1	2	0.001	EVM0013380 EVM0003562	Chemosensory protein precursor
1	2	0.024	EVM0008361 EVM0005241	Insulin-like growth factor 2 mRNA-binding protein
1	2	<1e-7	EVM0014740 EVM0000171	GTPase-activating protein pac-1-like
1	2	<1e-7	EVM0012402 EVM0000677	Hemolymph protein 14
1	2	0.001	EVM0013787 EVM0001757	Glutathione S-transferase
1	2	0.005	EVM0003715 EVM0006371	Hypothetical protein 3 - cabbage looper transposon TED
1	2	0.001	EVM0009126 EVM0008595	Retinol dehydrogenase 11-like
1	2	0.037	EVM0011934 EVM0013760	Apolipophorins
1	2	0.027	EVM0012995 EVM0001222	S-antigen protein
1	2	<1e-7	EVM0007537 EVM0012615	Cecropin A
1	2	<1e-7	EVM0004477 EVM0000882.	UDP-glycosyltransferase
1	2	<1e-7	EVM0007937 EVM0005030	Endonuclease and reverse transcriptase-like protein
1	2	0.023	EVM0014113 EVM0014355	Amino acid transport and metabolism; Serine protease 24
1	3	0.012	EVM0012395 EVM0001096	ATP-dependent DNA helicase MER
1	2	0.044	EVM0003209 EVM0014358	Proline-rich protein; lebocin-like protein
1	3	0.001	EVM0010172 EVM0008560 EVM0012239	Uncharacterized protein
1	2	<1e-7	EVM0001934 EVM0003728	Hypothetical protein 2 - cabbage looper transposon TED
1	2	<1e-7	EVM0009953 EVM0015050	Piggybac transposable element-derived
1	2	<1e-7	EVM0010118 EVM0011622	Receptor guanylate cyclase
1	2	0.014	EVM0006212 EVM0007033	Yolk protein 2
1	2	0.021	EVM0001362 EVM0000363	Uncharacterized protein
1	2	0.021	EVM0011788 EVM0007752	Pickpocket protein 28-like
1	3	<1e-7	EVM0000920 EVM0007834 EVM0004965	Uncharacterized protein

Other major expanded gene families were hemolymph protein [57], cecropin A [58], serine protease [59], apolipophorins [60], DNA helicase [61], insulin-like growth factor [62], and yolk proteins [63, 64] (Table 3). These gene families are supported to be involved in immunity, growth and development, biomacromolecule metabolism and reproduction in insects [57–59, 65–68].

### DEG analysis in different stages and tissues

**Fig. 2:**
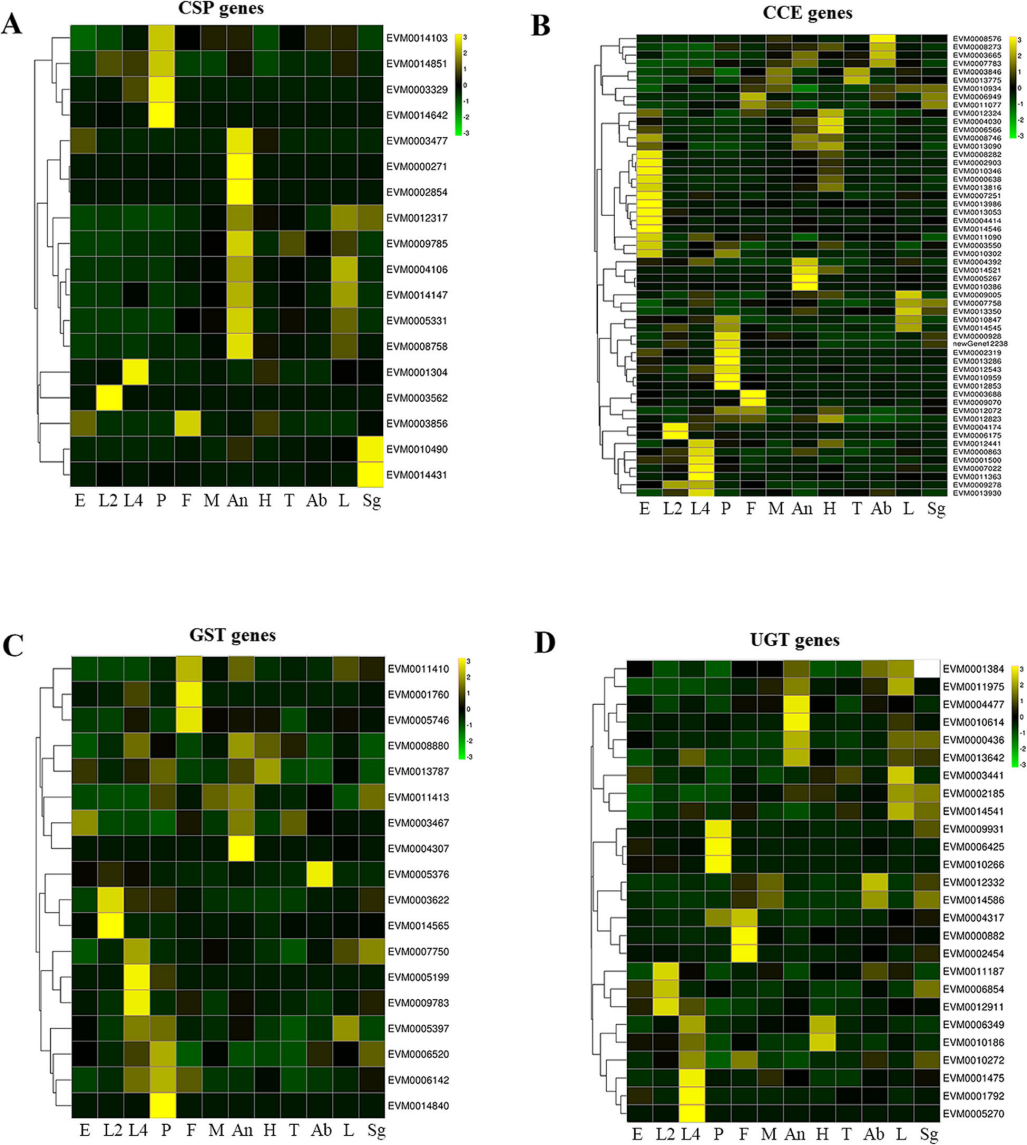
Expression heatmap of the expanded gene families in different tissues and stages. The colors represent the level of gene expression from low (purple) to high (yellow) as shown by log10 (FPKM + 0.000001). E (eggs), L2 (second-instar larvae), L4 (fourth-instar larvae), P (pupae), F (female adults), M (male adults); An (antenna), H (head), T (thorax), Ab (abdomen), L (leg), and Sg (female sexual gland). **a** Expression heatmap of the *CSP* gene family. The transcripts that were grouped together and specifically expressed in the antennae are *EVM0003477*, *EVM0000271*, *EVM0002854*, *EVM0002317*, *EVM0009785*, *EVM0004106*, *EVM0004147*, *EVM0005331* and *EVM0008758*. The transcripts that were highly expressed in pupae are *EVM0014103*, *EVM0014851*, *EVM0003329* and *EVM0014642*. The transcripts that were specifically expressed in the sex gland are *EVM0010490* and *EVM0014431*. **b** Expression heatmap of the *CCE* gene family. **c** Expression heatmap of the *GST* gene family. **d** Expression heatmap of the *UGT* gene family. The heatmaps were constructed with HemI (windows_1_0_win32_x64) using the DEGs of each expanding family. The expression levels are presented by the color bar from low expression as green with negative numbers and high expression level as yellow with positive numbers

To further study the chemosensory and detoxification gene families that were found to be expanded, transcriptome studies on these genes were performed to explore their expression profiles in different developmental stages and tissues. The analysis of differential gene expression by pairwise comparison led to the identification of 8232 DEGs whithn the different stages RNA (eggs, second instar larvae, fourth instar larvae, pupae, and male and female adults), and 7733 DEGs within the different tissues RNA (head, thorax, leg, abdomen, antenna, and female sexual glands). Then these two DEG datasets were combined, and the duplicated sequences were removed to create a final dataset of 10,348 DEGs (Table S7). The relative expression levels of these DEGs in different tissues and stages as indicated by log_10_FPKM values were shown in as the Box plot in Figure S6, and the numbers of alternative splicing events was showed in Figure S7. The expression of DEG gene families (*CSPs*, *GSTs*, *CCEs* and *UGTs*) was transformed into an expression heatmap and presented in Fig. 2 to better compare their expression levels in different tissues and developmental stages. Nine of the 20 *CSPs* were grouped together and specifically expressed in the antennae, four *CSPs* were highly expressed in pupae relative to other stages, while two *CSPs* were specifically expressed in the sex gland (Fig. 2a). In the expanded detoxification gene families *CCE*, *GST* and *UGT*, some genes were highly expressed in the fourth larval instar (Fig. 2b, c and d), which is the peak period of *H. cunea* foraging behavior [1].

### Positive selection on genes related to nutrient metabolism and detoxification

Next, a positive selection analysis based on the homolog genes was performed on the genome of *H. cunea* to gain a better understanding of the mechanisms in its host selection. The branch-site model showed that 39 genes were under significant positive selection pressure (LRT, *p* < 0.05), of which 13 were nutrient regulation genes reported to be involved in the metabolism of lipids, carbohydrates, vitamins and amino acids (Table 4). Many studies have shown that nutrient regulation in herbivorous insects is shaped by natural selection [69, 70]. Significant positive selection pressure was also detected in *HcunP450* (EVM0009687), a member of the major detoxification-related gene family *P450* (Table 4), consistent with a previous study reported that P450s could mediate insect resistance to many classes of insecticides [71]. *HcunP450* was most similar to the cytochrome *P450 CYP306A1* of the cotton bollworm *H. armigera* (AID54855.1), with 81.63% identity at the amino acid level. The expression of *HarmP450 CYP306A1* was found to be induced by 2-tridecanone, and to mediate cotton bollworm development [72]. The *CYP306A1* gene family was also shown to play an essential role in ecdysteroid biosynthesis during insect development [73], in fluoride resistance of *B. mori* [74]. Thus, the positive selection of *HcunP450 CYP306A1* might reflect the rapid development of insecticide resistance in *H. cunea*. However, it is needed to determine whether it is caused by long-term host adaptation or by rapid evolution due to the extensive use of insecticides in recent years.

**Table 4:** Genes under significant positive selection (LRT, *p* < 0.05)

Gene ID	***P***-value	Annotation
EVM0013226	2.79E-11	ADP-ribosylation factor 6
EVM0009669	2.58E-09	RNA-binding protein
EVM0012652	4.88E-09	Proteasome non-ATPase regulatory subunit
EVM0001215	2.12E-07	WD repeat domain-containing protein 83
EVM0000300	3.76E-05	Adenylosuccinate lyase
EVM0012350	0.000269395	Calcium uptake protein 1
EVM0013607	0.000594247	SNF-related serine/threonine-protein kinase
EVM0005265	0.000734493	Glycerophosphocholine phosphodiesterase GPCPD1
EVM0001021	0.0008973	Flavin reductase (NADPH)
EVM0012819	0.001467642	D-aspartate oxidase
EVM0014230	0.001971819	Cyclin-Y-like protein 1
EVM0010560	0.002186741	Ras-related protein Rab-32
EVM0008629	0.00253065	Pyridine nucleotide-disulfide oxidoreductase domain-containing protein 1
EVM0004149	0.005305157	Pentatricopeptide repeat-containing protein 1, mitochondrial
EVM0004370	0.006201581	Replication factor C subunit 3
EVM0001595	0.00637425	WW domain-binding protein 2
EVM0004769	0.00712973	Heparan-sulfate 6-O-sulfotransferase 2
EVM0004055	0.00764959	Transmembrane protein 147
EVM0000132	0.01233362	Carbonic anhydrase 1
EVM0004959	0.01293744	Phosphatidylinositol glycan
EVM0000082	0.01403601	Disco-interacting protein 2
EVM0015138	0.05027551	Activin receptor type-1
EVM0003028	0.15142045	Cyclin A
EVM0013889	0015549715	Phosphoserine aminotransferase
EVM0012220	0.015868726	Glycogen-binding subunit 76A
EVM0002611	0.016694366	E3 ubiquitin-protein ligase synoviolin A
EVM0005111	0.016757707	Prefoldin subunit 5
EVM0010778	0.018825687	Metaxin-1
EVM0006647	0.021983019	Vacuolar ATP synthase subunit E; V-type H + -transporting ATPase subunit E (A)
EVM0008673	0.024327862	Lysosome-associated membrane glycoprotein 1
EVM0000028	0.024646682	Transmembrane protein 183
EVM0010281	0.029467391	Secretion-regulating guanine nucleotide exchange factor-like
EVM0004884	0.030539908	Formin-binding protein 1-like
EVM0013117	0.034084621	Golgi apparatus protein 1-like
EVM0013726	0.03547635	Chitin binding domain protein
EVM0010443	0.036822765	3′(2′),5′-bisphosphate nucleotidase 1-like
EVM0010054	0.041270885	Very-long-chain enoyl-CoA reductase
EVM0009687	0.046760334	Cytochrome P450 306a1
EVM0002230	0.047306291	Ribosomal protein L27

### Compositional diversity of the gut microbiota

Our gut microbiota sequencing of *H. cunea* yielded 8.65 GB of valid data after filtering of *H. cunea* genome sequences and produced 28,846,959 clean reads and 151,448 contigs with a total length of 520.68 Mb after de novo assembly (Table S8). Based on the alignment of sequencing reads to the NCBI RefSeq database, the microorganism composition was annotated (Table S9) and analyzed, and the microbes were grouped into taxonomic categories from kingdom to species level. We found 324 kingdoms, 135 phyla, 13 classes, 244 orders, 157 families, 200 genera, and 78 species in the larval gut of *H. cunea*.

**Fig. 3:**
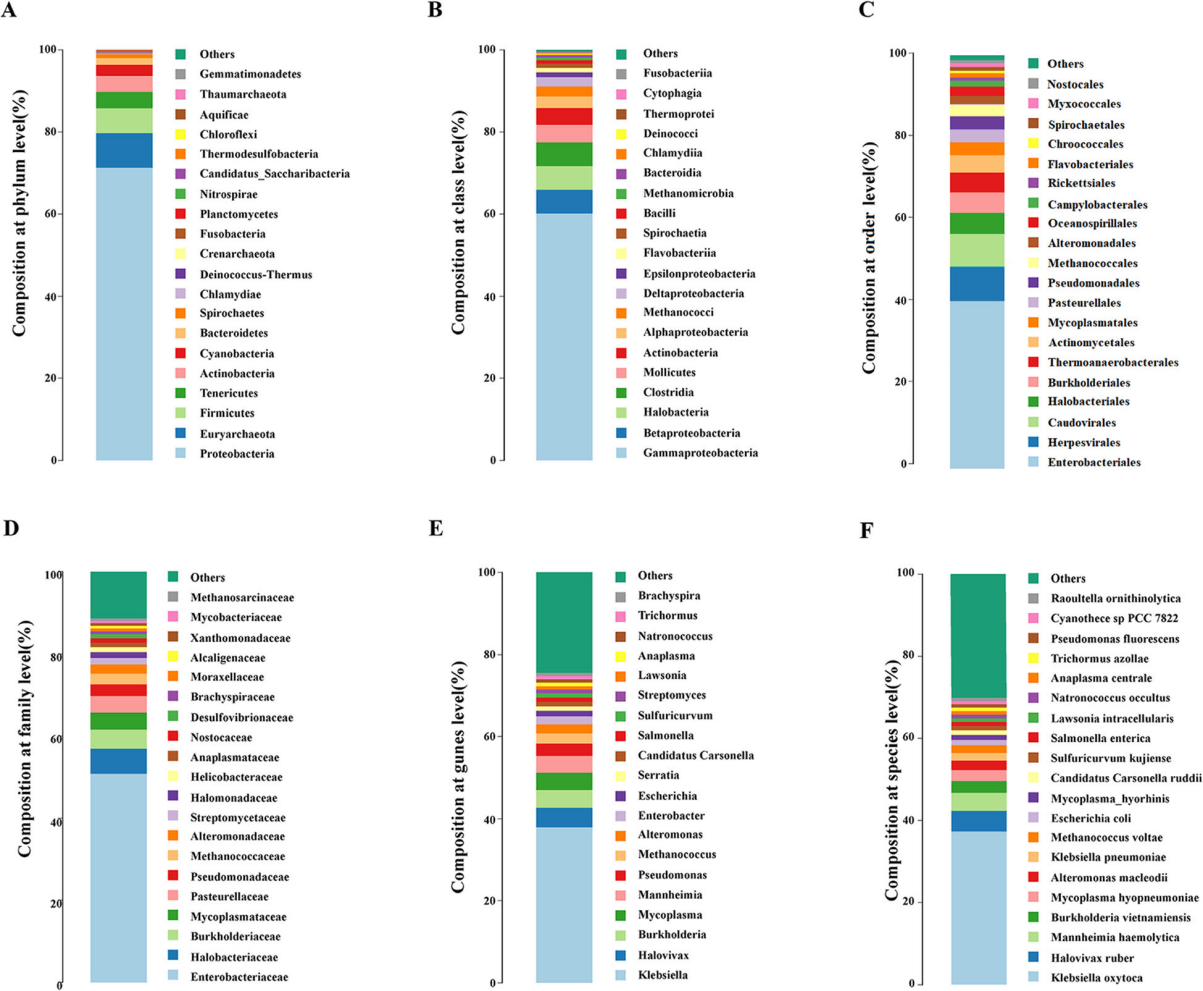
Taxonomic-category-based microbiota composition in the *H. cunea* larval midgut. **a** Microbiota proportion at the phylum level. **b** Microbiota proportion at the order level. **c** Microbiota proportion at the class level. **d** Microbiota proportion at the family level. **e** Microbiota proportion at the genus level. **f** Microbiota proportion at the species level

At the phylum level, the *H. cunea* gut microbiota was dominated by Proteobacteria (71.33% of the total midgut bacteria contigs), followed by Euryarchaeota and Firmicutes (8.40 and 6.10% of the contigs, respectively) and to a lesser extent, Tenericutes, Actinobacteria, Cyanobacteria and Bacteroidetes; other phyla were less than 1% of the total contigs (Fig. 3a). At the class level, Gammaproteobacteria, Betaproteobacteria, Halobacteria and Clostridia comprised 77% of the contigs (Fig. 3b), while Enterobacteriales, Halobacteriales and Burkholderiales comprised 60% of all contigs at the order level (Fig. 3c). The three most abundant families were Enterobacteriaceae, Halobacteriaceae and Burkholderiaceae (50.86, 6.16, and 4.58% of total contigs, respectively) (Fig. 3d). At the genus level, microorganisms were rich in *Klebsiella*, *Halovivax* and *Burkholderia* (37.92, 4.75 and 4.32% of total contigs, respectively) (Fig. 3e). *Klebsiella oxytoca* was the most abundant species in the midgut of *H. cunea*, followed by *Halovivax ruber*, *Mannheimia haemolytica*, and *Burkholderia vietnamiensis* (Fig. 3f).

### Functional annotation of the leaf-eating caterpillar gut metagenome

Our metagenomic analysis led to the identification of 102,787 nonredundant protein-coding genes with an average length of 300 bp (30.80 Mb total length) in the microbiota of the *H. cunea* larval gut. Gene functional annotation based on KEGG pathways showed that the most abundant function in the metagenome was metabolic function, representing 45.16% of all KEGG functions in the *H. cunea* gut microbiota.

**Fig. 4:**
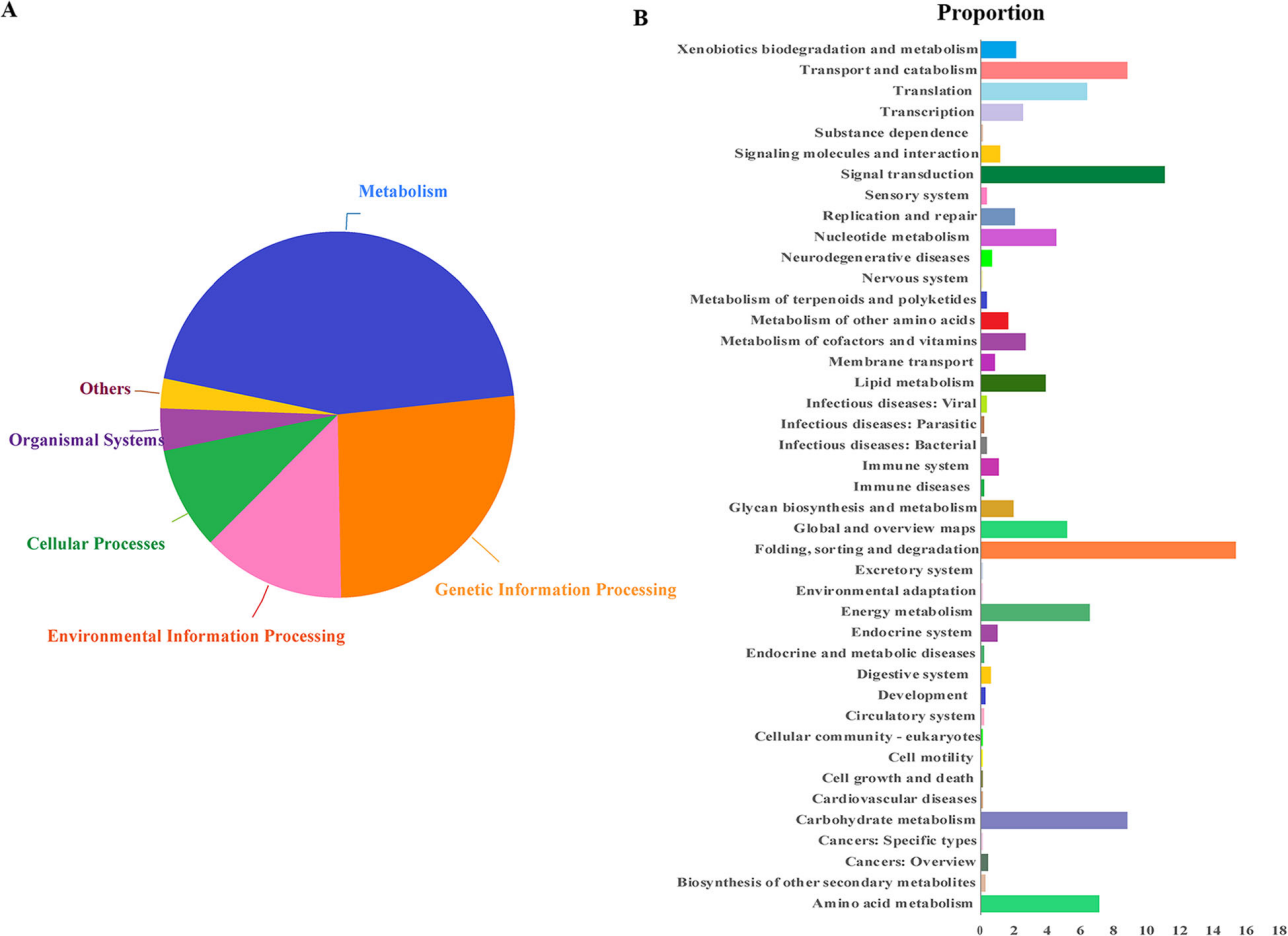
Metagenomic genes in the midgut of *H. cunea* larvae. **a** KEGG pathway functional annotations of metagenomic genes. **b** Functional enrichments of metagenomic genes. The X-axis indicates the proportion of genes annotated in each category

KEGG iPath 2 analysis showed that the metabolic activities of the *H. cunea* gut bacteria were associated with digestion, nutrition and detoxification, including metabolism of energy, carbohydrates, amino acids, lipids, cofactors, vitamins, glycans, xenobiotics, and terpenoids. The most enriched functions within these activities were “Folding, sorting and degradation”, representing 15.35% of all KEGG pathways, followed by “Signal transduction” (11.08%). The nutrient metabolism functions that could be provided by gut microbiota were “Carbohydrate metabolism” (8.83%), “Amino acid metabolism” (7.09%), “Energy metabolism” (6.59%), “Nucleotide metabolism” (4.55%), “Lipid metabolism” (3.90%), “Glycan biosynthesis and metabolism” (1.93%) and “Metabolism of cofactors and vitamins” (2.72%). In addition, genes in the gut microbiota were found with functions related to “Xenobiotics biodegradation and terpenoid metabolism” (2.09%) and “Biosynthesis of other secondary metabolites” (0.31%) (Figs. 4 and S9 and Table S10).

A total of 336 enzymes associated with cellulose and hemicellulose hydrolysis were identified in the intestinal flora of *H. cunea* based on the Carbohydrate-Active EnZyme (CAZy) database, including 42 auxiliary activities (AAs), 68 carbohydrate binding modules (CBMs), 75 carbohydrate esterases (CEs), 68 glycoside hydrolases (GHs), 82 glycosyltransferases (GTs) and one polysaccharide lyase (PL) (Figure S8 and Table S11). The results indicate that the gut microbes of *H. cunea* were most likely involved in cellulose degradation. By sequence alignment, we also predicted 55, 256 and 236 genes possibly encoding for glutathione S-transferases, esterases, and P450*s*, respectively (Table S12).

### Silk-web-related genes

Notably, one gene family related to silk production, Kazal-type serine proteinase inhibitors (*KSPIs*) [75], showed an expansion among the tested orthologous gene groups, which implies that silk-related genes might also have a role in the environmental adaptation to larval development of *H. cunea*. Hence, we performed further studies on silk-web-related genes.

**Fig. 5:**
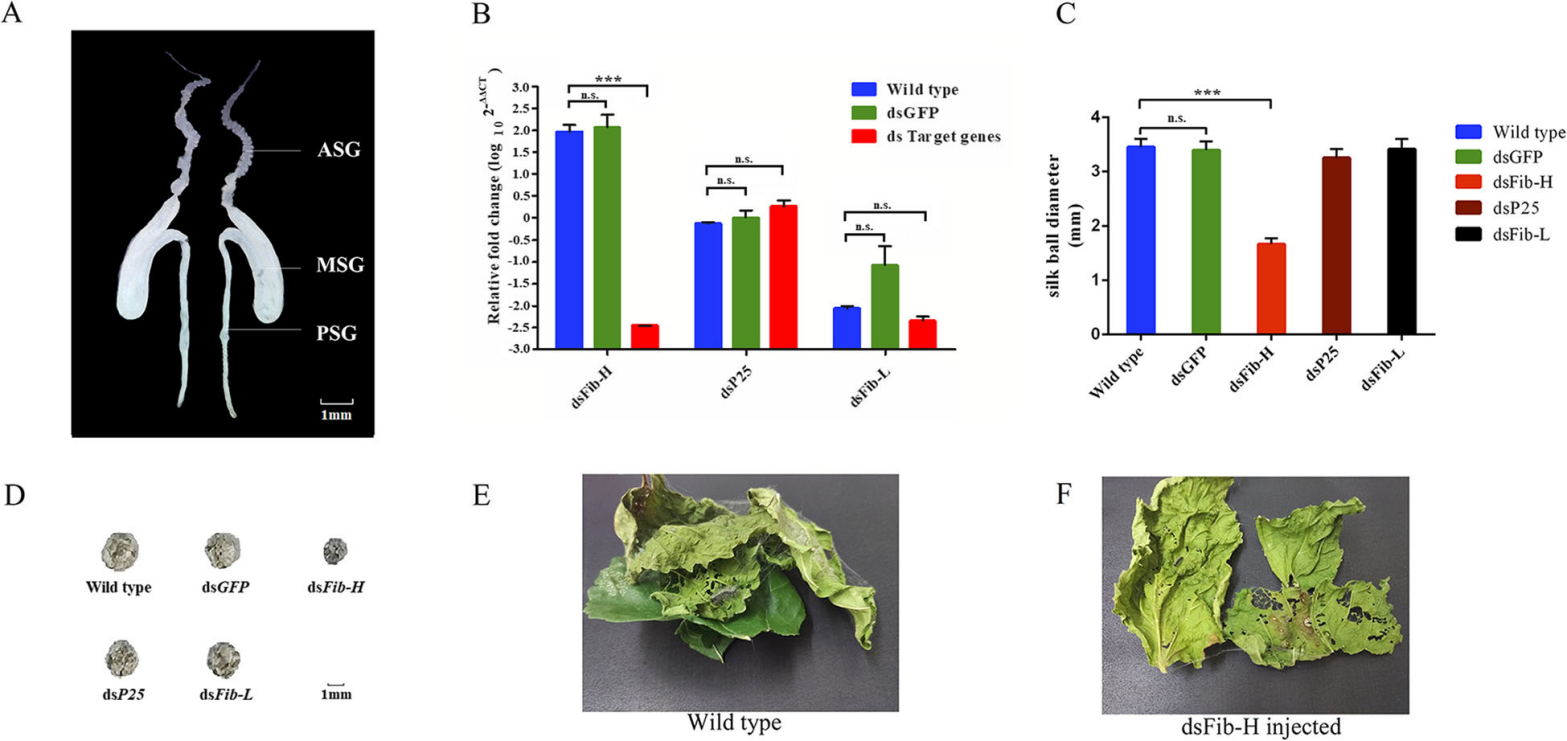
Silencing results of *HcunFib-H* and phenotype analysis. **a** Anatomy of the larval silk gland of *H. cunea*. ASG indicates anterior silk gland, MSG indicates middle silk gland and PSG indicates posterior silk gland. **b** RT-qPCR results 4 days after dsRNA injection for RNAi. Statistical differences were evaluated by t-tests. *** *p* < 0.001, n.s., not significant. Data indicate the means + SEM, *N* = 5. The expression levels were normalised by the expression of β-actin gene in different treatment samples. **c** & **d** Diameter of the silk ball after RNAi; statistical differences were evaluated by t-tests. *** *p* < 0.001, n.s., not significant, scale bar = 0.5 mm. **e** & **f** Comparison of silk spinning between wild-type and ds*HcunFib-H*-injected insects

The silk gland is a long paired organ of the fall webworm. It specializes in the synthesis and secretion of silk proteins (Fig. 5a) and quickly atrophies after the onset of adulthood. The anatomy of the silk gland in the fall webworm is quite similar to that of *B. mori*, and consists of three functionally distinct regions: the anterior silk gland (ASG), middle silk gland (MSG) and posterior silk gland (PSG) [76]. Thirty-three silk-gland-related genes were identified in *H. cunea* (Table 5) through a homologous search against those from other Lepidopteran silk glands in previous studies [76–78], including 3 silk protein genes, 4 silk regulation genes and 26 protease inhibitor genes. In *B. mori*, the silk protein is composed of a ((Fib-H) - (Fib-L))_6_ -P25 fibroin complex and held together by the protein sericin [79]. Here, three fibroin structure genes, *HcunFib-H*, *HcunFib-L* and *HcunP25*, were identified from the *H. cunea* genome, but our results showed that no sericin genes were annotated in the *H. cunea* genome; however, some silk regulation genes, such as silk gland factors (*SGFs*), fibroin-modulator-binding protein-1 (*FMBP-1*) and fibroinase, were identified in the *H. cunea* genome. Moreover, several protease inhibitors, such as kazal-type serine protease inhibitors, pacifastin-related serine protease inhibitor (pacifastin), phosphatidylethanolamine-binding protein, alpha-2-macroglobulin (A2M), cysteine proteinase inhibitor, carboxypeptidase inhibitor, cystatin, serpins and proteasome inhibitor genes, were identified.

**Table 5:** Identification of silk-web-related genes

Functional classification	Gene name	Gene ID
Silk protein genes	Fibroin light chain gene (*Fib-L*)	EVM0009358
	Fibroin heavy chain gene (*Fib-H*)	EVM0005282
	25 kDa silk protein (*P25*)	EVM0009847
	Sericin gene	None
Silk regulate genes	Fibroinase	EVM0000430
	Fibroin-modulator-binding protein-1	EVM0012647
	Silk gland factor	EVM0004972; EVM0012444
Protease inhibitor	Kazal-type serine protease inhibitor (*kazal*)	EVM0003147;EVM0009039;EVM0001538;EVM0000158; EVM0015003;EVM0006856; EVM0006205;EVM0013167; EVM0000098
	Pacifastin-related serine protease inhibitor (pacifastin)	EVM0013021
	Phosphatidylethanolamine-binding protein (*PBP*)	EVM0007560;EVM0006444; EVM0011908;EVM0008732; EVM0002652;
	Alpha-2-macroglobulin (*A2M*)	EVM0001565; EVM0002258
	Cysteine proteinase inhibitor	EVM0003884;EVM0006961; EVM0010793;EVM0002065; EVM0006398
	Carboxypeptidase inhibitor	EVM0003948
	Cystatin	EVM0010793; EVM0012410
	Proteasome inhibitor	EVM0011839

### Silencing of silk fibroin genes and phenotype analysis

Because silk is a structural material and plays a crucial role in the survival of many insects, the extraordinary mechanical properties of silk are often explained in adaptive terms [80]. Fibroin is the key component of silk; it determines both the quantity and the structure of a silk web [81]. Here, three structural protein genes, *HcunFib-H*, *HcunFib-L* and *HcunP25*, were chosen for RNAi experiments to explore the mechanism of web production in *H. cunea* because of their involvement in silk production. We targeted three fibroin genes for silencing and measured their expression levels by qRT-PCR 4 days after injection (Fig. 5b). In comparison with the noninjected groups, there were no significant changes in the expression of *GFP*, *HcunFib-L* and *HcunP25* (*p* > 0.05), while the relative expression of *HcunFib-H* was dramatically decreased (*p* < 0.001). The different expression levels among the three genes might be one of reasons that resulted in the differences in RNAi as the expression of *HcunFib-H* was much higher than those of *HcunFib-L* and *HcunP25.*

Within 10 days after the injection, the average diameters of the silk balls in the different treatments were as follows: that of the noninjected wild type was 3.45 ± 0.12 mm; ds*GFP*-injected, 3.39 ± 0.14 mm (*p* = 0.64 > 0.05); ds*HcunFib-L*-injected, 3.41 ± 0.15 mm (*p* = 0.79 > 0.05); ds*HcunP25*-injected, 3.25 ± 0.14 mm (*p* = 0.18 > 0.05) and ds*HcunFib-H*-injected, 1.67 ± 0.09 mm (*p* < 0.0001) (Fig. 5c and d). There was a significant decrease in the quantity of silk in the ds*HcunFib-H* injected group, which was consistent with the dramatic decrease in the gene expression of *HcunFib-H* of the ds*HcunFib-H-*injected group after RNAi. The silencing of the silk structure protein gene *Fib-H* led to less silk production and damaged the leaf-silk shelter structure of the fall webworm by breaking the silk-leaf connections (Fig. 5e and f), suggesting that *HcunFib-H* contributes significantly to the formation of fibroin, to related web-producing behaviors and to the silk-web-related adaptations of *H. cunea*.

## Discussion

In this study, the genome of the fall webworm we obtained was of high integrity by PacBio sequencing. And compared with other publicly available Lepidopteran genomes (*Plutella. xylostella*, *Papilio polytes*, *Papilio machaon*, *Papilio xuthus*, *Pieris rapae*, *H. armigera*, *O. brumata*, *B. mori*, *S. frugiperda* and *P. bianor*), the *H. cunea* genome possesses a comparatively longer contig N50 (only smaller than the genome of *O. brumata*, *S. frugiperda* and *P. bianor*). The large genome size of *O. brumata* could be explained to a large extent by its higher repeat content, containing 53.5% repetitive elements in *O. brumata* genome (35.7% in *H. cunea* and 38.4% in *B. mori* genomes) [40]. However, the large genome of *H. cunea* is more likely to be caused by a larger average intron size, the mechanism is worthy of further study, because the average intron size of the *H. cunea* genome was 1491 bp, much larger than 1082 bp of *B. mori* and 139 bp of *O. brumata*. A similar phenomenon was also reported in the *Locusta migratoria* genome [82].

According to the result of the phylogeny of Lepidoptera, *H. cunea* and *H. armigera* were estimated to have diverged at the Eocene-Oligocene boundary, while from the late Eocene to early Oligocene, with the end of a continuous cooling event [83], deciduous trees that were better able to cope with large temperature changes began to overtake evergreen tropical species [84]. In North America, where *H. cunea* is native, litchi and cashew nut were the dominant trees in the early Oligocene [85] . With the expansion of temperate deciduous forests during this epoch, the food sources of the fall webworm increased, which might have contributed to the expansion of the host range of *H. cunea*.

The *CSP*, *CCE*, *GST* and *UGT* gene families were expanded in *H. cunea* compared to the tested Lepidopteran species, similar expansions of the chemosensory gene family have also been detected in other insect genomes [13, 18, 86, 87]. For example, studies of 22 mosquito species found that a distinct clade of *CSPs* was expanded in three *Culicinae* species [86], and a lineage-specific expansion was present in the whitefly *Bemisia tabaci* compared with *Adelphocoris lineolatus*, *Aphis gossypii*, *Apolygus lucorum*, *Myzus persicae*, *Nilaparvata lugens* and *Sogatella furcifera* [18]. However, additional tests are still needed to determine whether these *CSPs* respond to host plant volatiles of *H. cunea*. Many studies have shown that *GSTs* and *CCEs* mediate insect tolerance to allelochemicals and contribute to resistance to a wide range of insecticides [88, 89], while *UGTs* play a crucial role in detoxification and in the regulation of xendobiotics in insects [90]. Previous studies of *Culex quinquefasciatus* and *Aedes aegypti* reported that the expansion of detoxification genes might involve in making these insects particularly adaptable to polluted water and contribute to their development of metabolic resistance to pyrethroid insect pesticides [91]. Therefore, the expansion of detoxification genes in *H. cunea* might also reflect their wide range of host plants thus adaptation by enhancing their capacity to detoxify xenobiotics and resist insecticides [92]. For other major expanded gene families, the hemolymph protein has been studied as an antifreeze protein, contributing to insect cold adaptation [93]; cecropin serves as an antibacterial protein of the insect immune system, supporting resistance to pathogenic microorganisms, and might be responsible for the adaptation of living organisms to environmental conditions [94]; serine proteases are known to dominate the lepidopteran larval gut environment and contribute to the polyphagous nature of insect pests such as *H. armigera* [95]*.* However, more experimental and field tests are needed to determine if the expansion of these three gene families could play important roles in the adaptation of *H. cunea* to environmental changes.

The nine *CSPs* were grouped together and specifically expressed in the antennae, strongly suggesting roles in olfactory sensing and host location. While the six *CSPs* expressed in pupae or sex gland might have other biological functions in *H. cunea* rather than those relating to olfaction such as carbon dioxide detection, larval development and leg regeneration reported previously [96] . For detoxification genes, in the lepidopteran *S. frugiperda*, detoxification genes such as *CCEs* and *GSTs* were much more highly expressed in lufenuron-resistant larvae than in lufenuron-susceptible larvae [97]. Some detoxifying genes in *Heliconius melpomene* larvae, including those encoding for GSTs, UGTs or P450s, responded significantly to a host plant shift [98]. In addition to lepidopteran insects, studies of other polyphagous species, *Tetranychus urticae* and *Anopheles gambiae*, also showed that the expression levels of many detoxification genes were significantly increased in association with host plant shifts or feeding stages [99, 100]. Furthermore, insect larvae are exposed to a range of food sources thus plant allelochemicals and a variety of bacterial toxins. The ability of insect species to tolerate these toxins can influence their distribution [101]. These findings suggest that the detoxification genes that were highly expressed during the peak feeding period of *H. cunea* might contribute to *H. cunea* host plant adaptation or host shift [102]. Additionaly, the positive selection of *HcunP450 CYP306A1* might reflect the rapid development of insecticide resistance in *H. cunea*, but much more testing is required.

There is growing evidence that the gut microbiota of insects plays crucial roles in diverse functions for the hosts, including growth, development and environmental adaptation [25]. Metagenomic approaches have been successfully applied to understand the relationship between gut microbiomes and their hosts over the past decade [103, 104]. The results of our study showed that the predominant phyla of bacteria in the gut of *H. cunea* larva were Proteobacteria and Firmicutes (Fig. 3a). Similar results have been found in previous studies of many different orders [25, 105]. These two phyla are also ubiquitous in the guts of lepidopteran insects such as *H. armigera* [106] and *B. mori* [107]. Proteobacterial symbionts are considered to be useful to the digestion of host insects [108] and to be involved in carbohydrate degradation and nitrogen fixation [109, 110], which help their hosts prevent the establishment and proliferation of pathogenic bacteria [111–114]. Firmicutes play a role in insecticide degradation and increase the abundance of resistant lines [26, 110, 112] . The three most abundant microbes in *H. cunea* gut at the genus level were *Klebsiella* (37.92%), *Halovivax* (4.75%) and *Burkholderia* (4.32%) (Fig. 3e); these results are very different from those of the gut microbiome of the host specialist *B. mori*. In the gut microbiota of *B. mori* (a standard inbred strain, Dazao), *Enterococcus* (18.7%), *Acinetobacter* (16.20%) and *Aeromonas* (8.70%) were the three most abundant microbial genera [115]. The genera *Klebsiella*, *Halovivax* and *Burkholderia* that occur in *H. cunea* have been reported to contribute to cellulose degradation [116], nitrogen fixation [117], carbon metabolism [118, 119], insect growth [120] and fenitrothion resistance [121]. Thus, the abundance of these bacteria might imply their contribution to host adaptation in *H. cunea* [122–124], but much more testing is required. Moreover, the carbon in plant cell walls exists in the form of cellulose, hemicelluloses, and lignin and is largely inaccessible to most organisms [125]. It is now well understood that gut symbiotic communities, most notably the symbiotic bacteria of termites [126] and ruminants [127], play a pivotal role in cellulose deconstruction in many invertebrates and vertebrates. Thus, the functional annotation of the leaf-eating caterpillar gut metagenome of the fall webworm was studied.

The gregarious larvae of *H. cunea* build conspicuous leaf-silk shelters on their host trees [128], where the larvae feed and live as they grow. The fall webworms generally aggregate in the web during daylight and extend their webs at night to enclose edible leaves for feeding [34, 129]. Moreover, this extended web can provide a protected space for larval development by blocking environmental damage or attacks from natural enemies, and is used as a support during ecdysis [130]. The silk web also regulates heat and slows air movement within the web, fostering the development of *H. cunea* larvae [34, 131]. The temperature inside the webs is considerably higher compared to the ambient temperature, and the interior heat-retention properties of the web rely mainly on the thickness, abundance and color of the web than on behavioral factors [34]. These physicochemical characteristics of the silk web are modified by the silk proteins [132]. Althought, the anatomy of the silk gland of *H. cunea* is quite similar to that of *B. mori*, the composition of the silk protein between *H. cunea* and *B. mori* was different. In our case, there were no sericin genes identified from the genome and 12 transcriptome datasets of *H. cunea*. For sericin, some studies have shown that the sericin-related protease inhibitor in *B. mori* functions to protect the silk web or cocoon from degradation [133–137], but these glue-like proteins are absent in some spider species [138]. And some saturniid insects lack Fib-L and P25 proteins in the fibroin complex [130]. The silk contains 70%~ 75% fibroin [139], of which Fib-H accounts for 93% (w/w) of the composition in Lepidoptera [140]. To explore the function of the silk structure proteins in *H.cunea*. The three silk protein genes were selected from the 33 silk gland related genes to RNAi test. The result of RNAi suggesting that the *HcunFib-H* plays a critical role in the formation of fibroin, the web-producing behaviors and the silk-web-related adaptations of *H. cunea*. This gene could potentially be used as a target for future pest management of *H. cunea*.

For silk regulation genes, there is conclusive evidence showing that these genes are involved in regulating the synthesis or degradation of sericin and fibroin. *SGFs* stimulate the transcription of *sericin-1* via different binding sites [141, 142] and play a key role in regulating tissue-specific expression of the fibroin gene [143]. *FMBP-1* regulates the specificity of fibroin gene expression by binding the upstream and intronic promoter elements of the fibroin gene [144, 145]. Fibroinase in the silk gland is a cathepsin L-like cysteine proteinase that can digest silk proteins in the lumen of the silk gland after spinning and is regulated by protease inhibitors [146]. For protease inhibitors, many studies have shown that some protease inhibitors are specifically expressed in silk glands to avoid infection [77, 147]. The expanded *KSPI* gene family, as a silk proteinase inhibitor 2 (SPI 2) in Lepidoptera, was reported to be involved in the inhibition of bacterial subtilizing and fungal proteinase K activity [75].

## Conclusion

The first genome of the worldwide invasive pest *H. cunea* was obtained, and further studies revealed three causes of *H. cunea*’s adaptation. First, some chemosensory and detoxification gene families were expanded, suggesting the contribution of these genes to their extreme polyphagy at a genomic level. In addition, several nutrient metabolic and detoxification genes were found to evolve more rapidly along with their host expansions. Second, our results support that some gut microbes and their metabolic pathways are able to assist their nutrient metabolic and detoxification and might be involved in the host adaptation of *H. cunea*. Third, the silk web, which has been shown to function in the aggregated foraging and thermal regulation behavior of *H. cunea*, was further explored by silencing one of the silk protein gene *HcunFib-H*, significantly decreasing the quantity of silk and breaking silk-leaf connections. Overall, our results provide some evidence on the adaptation of *H. cunea*, partially explaining the reasons for the rapid invasion of *H. cunea* at the genome, transcriptome and metagenome levels, along with some potential gene targets and innovative strategies for the control of this invasive pest.

## Methods

### Insects

A colony of *H. cunea* was established from a single egg mass and maintained in our laboratory for population expansion to reduce heterozygosity. Because low genomic heterozygosity is important for obtaining high-quality genomes, we used a fifteen-generation inbred population of *H. cunea* from a single egg mass. The egg mass was collected in the field from damaged forest in Beiling Park, Liaoning Province, China, in 2015. The colony was fed fresh mulberry leaves (26 °C, 80% RH, 19:5 light:dark cycle, BIC-300 artificial climate chest, Boxun, Shanghai, China). Genomic DNA was extracted using the cetyltrimethylammonium bromide (CTAB) method from a single adult male. The sample was washed with double-distilled water and frozen in liquid nitrogen before DNA extraction. After measuring the concentration and quality, the genomic DNA was immediately stored in a − 80 °C freezer until further sequencing.

### Genome sequencing and assembly

First, we performed a preliminary survey to evaluate the genome size, repeat sequence ratio and heterozygosity of the *H. cunea* genome; for this, a genome survey with k-mer analysis was used as a general and assembly-independent method for estimating these three genomic characteristics as meantioned above, the 270 bp library data were used to construct a k-mer distribution map for k = 19. Then, genome sequencing was performed on two separate platforms (PacBio and Illumina). For PacBio genome sequencing, the genomic DNA was sheared using g-TUBE devices (Covaris, Inc., USA) and purified using a 0.45 volume ratio of AMPure PB beads. SMRTbell libraries were created using the ‘Procedure & Checklist—20 kb Template Preparation using BluePippin™ Size Selection’ protocol [148]. The quality of the library was tested with a Qubit fluorometer (Invitrogen Life Technologies, CA, USA) and an Agilent 2100 Bioanalyzer (Agilent Technologies, Santa Clara, CA, USA). Then, the library was sequenced on a PacBio RSII (Expression Analysis, Durham, NC, USA) platform. For Illumina sequencing, two libraries with insert sizes of 270 bp and 500 bp were built with the Ultra II DNA Library Prep Kit (New England Biolabs, Ipswich, MA, USA) and sequenced by using the Illumina HiSeq X Ten system (Illumina, San Diego, CA, USA) with rapid runs. All sequencing was conducted by Biomarker Technology Co., Ltd. (Beijing, China).

The sequencing adapters and the low-quality reads (with read quality score < 50 or length < 50 bp) in the sequencing reads were removed by the RS Subreads Protocol of SMRT Analysis version 2.3 [149]. We corrected the PacBio reads with Canu version 1.7 [150] and then assembled the retained high-quality subreads with Canu v1.5, Falcon v0.7 and WTDBG v1.2.8 [151, 152] independently. Finally, the draft assembly was corrected and polished with Pilon [153] using high-coverage Illumina reads. Based on the optimal assembly results, we evaluated the completeness of the genome assembled by WTDBG. The alignment efficiencies were calculated by mapping the reads generated by the Illumina platform and the reads corrected by Canu to the assembled genome. Then, two databases, the Core Eukaryotic Genes Mapping Approach (CEGMA v2.5) [154] and Benchmarking Universal Single-Copy Orthologs (BUSCO v2.0) [155], were used to assess the completeness of the WTDBG assembly.

### Repeats and noncoding RNAs

The specific repetitive sequence database was used to predict repeat sequences. A de novo repeat library of *H. cunea* was constructed by LTR_FINDER v1.05 [156], MITE-Hunter [157], RepeatScout v1.0.5 [158] and PILER-DF v2.4 [159]; then, it was classified by PASTE Classifier [160] and combined with the Repbase transposable element library to act as the final library. Afterward, RepeatMasker v4.0.6 [161] was used to find the homologous repeats in the final library. tRNAscan-SE v1.3.1 [162] was used to search for tRNA coding sequences. rRNA and microRNA were identified by Infernal v1.1 [163] based on the Rfam database and miRBase database. The pseudogene were predicted by two steps: Firstly, GenBlastA v1.0.4 [164] was applied to identify the candidate pseudogene by homologous searching against genome data. Secondly, GeneWise v2.4.1 [165] was performed to search for immature termination and frameshift mutation of pseudogene .

### Gene prediction and functional annotation

To identify protein-coding sequences, a combination of ab initio gene prediction, homology-based prediction and unigene-based methods were used as annotation pipelines. Genscan [166], Augustus v2.4 [167], GlimmerHMM v3.0.4 [168], GeneID v1.4 [169] and SNAP [170] were used to predict the protein-coding sequences. Four species (*Amyelois transitella*, *Bombyx mori*, *Helicoverpa armigera* and *Plutella xylostella*) were used to complete homology-based gene prediction with GeMoMa v1.3.1 [171]. Reference transcriptome assembly was performed by HISAT v2.0.4 and StringTie [172], and gene prediction was performed by TransDecoder v2.0 [173] and GeneMarkS-T v5.1 [174]. The de novo transcriptome was completed by PASA v2.0.2 [175]. Finally, all the results from three gene prediction methods (GeMoMa, TransDecoder v2.0 and GeneMarkS-T) were integrated by EVidenceModeler (EVM) v1.1.1 [176] and annotated by PASA v2.0.2.

Gene functions were assigned according to the best-match BLASTp alignments in the NR databases, KOG, TrEMBL and Kyoto Encyclopedia of Genes and Genomes (KEGG). GO annotations were obtained by Blast2GO based on the results of alignment to NR. Moreover, we also performed enrichment analyses of the Clusters of Orthologous Groups of proteins (COG), GO terms and KEGG pathways.

### Orthologous gene families

The most updated genome sequences of twelve sequenced insects (*Apis mellifera*, *Bombyx mori*, *Drosophila melanogaster*, *Helicoverpa armigera*, *Papilio machaon*, *Papilio polytes*, *Papilio xuthus*, *Pieris rapae*, *Plutella xylostella*, *Tribolium castaneum*, *Hyphantria cunea*, and *Operophtera brumata*) were used to infer gene orthology and construct the phylogenetic tree, the details of these genomic datasets we used in this study were showe in Table S13. After downloading the annotated coding sequences from NCBI, the longest protein sequences per gene were extracted to perform a best reciprocal hit (BRH) analysis by all-v-all BLAST using an E-value equal to 1E− 05 to identify orthologous genes among the twelve species by OrthoMCL 5 [177].

### Phylogenetic tree and divergence times

The longest open reading frames (ORFs) for the longest transcript pairs across the twelve species were extracted by a Perl script, and tORFs in each orthologous set were aligned using PRANK [178] with the following parameters: −f = fasta -F -codon -noxml -notree -nopost. The alignment for each locus was trimmed by Gblocks v 0.91b [179] (Parameters: −t = c − b3 = 1 − b4 = 6 − b5 = n) to reduce the rate of false positive predictions by filtering out sequencing errors, incorrect alignments and no-orthologous regions based on codons [180]. After trimming, alignments of less than 120 bp were removed. The single-copy orthologous genes were concatenated into one supergene, and the best amino acid substitution model was estimated. RAxML v. 8.0.26 [181] was used to construct the phylogenetic tree based on the supergene under the LG + I + G + F model with 1000 bootstrap replicates. The divergence times among species were estimated by R8s v. 1.7.1 [182] with a node dating approach that used three fossil records as the most recent common ancestor. The three fossil records we used in this study were the oldest definitive beetle (Coleopsis archaica gen. et sp. Nov., 298.9 to 295.0 Ma), the oldest fossil Diptera (such as: Anisinodus crinitus n. gen., n. sp., 247.2 to 242.0 Ma) and the oldest fossil Rhopalocera (Praepapilio Colorado n. g., n. sp., *P. gracilis* n. sp., and Praepapilioninae. Riodinella nympha n. g., n. sp., 46.2 to 40.4 Ma), respectively [183–185].

### Gene family expansion/contraction

CAFE [186] was used to examine the expansion and contraction of gene families among the twelve species. The results of the orthologous gene identification were filtered by CAFE’s built-in script, and the global parameter λ was estimated by the maximum likelihood method. Comparing divergence size and species size calculated by CAFE could determine whether expansion had occurred. The divergence size indicates the ancestral gene family size for each node in the phylogenetic tree, and the species size indicates the gene number in the homologous gene family. When the divergence size is smaller than the species size, the gene family is expanding. Additionally, for each gene family, a conditional *P*-value was calculated, and gene families with *P*-values < 0.05 were considered to have significantly expanded or contracted.

### Positive selection analyses

A branch-site model (parameters: Null hypothesis: model = 2, NSsites = 2, fix_omega = 1, omega = 1; alternative hypothesis: model = 2, NSsites = 2, fix_omega = 0, omega = 1) in PAML [187] was used to identify the genes with positively selected sites in the fall webworm genome using our tree topology as the guide tree. Then, likelihood ratio tests (LRTs) were performed to detect positive selection on the foreground branch. Only those genes with LRT *P*-values less than 0.05 were inferred as positively selected.

### Transcriptome analysis of different stages and tissues

RNA sequencing was performed on different developmental stages and tissues of *H. cunea*. The following developmental stages were selected for the transcriptome analyses: eggs, second instar larvae, fourth instar larvae, pupae, and male and female adults. The following tissues were used for the tissue transcriptome experiment: head, thorax, leg, abdomen, antenna, and female sexual glands. For each group, fifteen individuals were mixed for RNA extraction, and three biological replicates were produced for each sample. Total RNA was isolated from the homogenized samples using TRIzol reagent (Invitrogen, Carlsbad, CA, USA) according to the manufacturer’s protocols. After extraction, total RNA was assessed with the NanoDrop 2000 (Thermo Fisher Scientific, Waltham, MA, USA) and the Agilent Bioanalyzer 2100 System (Agilent Technologies, CA, USA) to verify the integrity and quality of RNA.

After each sample was quantified, the libraries were built and sequenced on the Illumina HiSeq X Ten platform. After filtering, clean reads were mapped to the reference genome sequence obtained in this study with Hisat2 tools [188]. Only reads with a perfect match or one mismatch were retained for further analysis. Cufflinks counts the expression of each gene and reports it in fragments per kilobase of transcript per million fragments mapped (FPKM) [189]. For each sequenced library, the read counts were adjusted by the edgeR package [190] with one scaling normalized factor. Differentially expressed gene (DEG) analysis within two sample groups (stages and tissues) was performed using the EBSeq R package [191], and then the false discovery rate (FDR) was performed based on the Benjamini-Hochberg (BH) procedure [192] to correct the *P* value of the identified datasets, with the standard of FDR ≤ 0.01 and fold change (FC) ≥ 2 to remove the false positive datasets.

### Metagenomic sequencing and analysis

To test whether symbiotic microbes facilitate environmental adaptation in *H. cunea*, detailed profiles of the gut microflora were obtained by metagenomic sequencing. Midgut samples were collected from ten last-instar larvae of *H. cunea* from the same wild population on their host plant (*Quercus mongolica*) and preserved in RNAlater. DNA was extracted from a mixture of ten gut samples using an effective gut microbiota DNA extraction kit (QIAamp DNA Stool Mini Kit; Qiagen) and stored at − 20 °C. A paired-end gut microbiota DNA library was built using the NEBNext DNA Library Prep Mast Mix Set for Illumina (New England Biolabs, Ipswich, MA, USA). Sequencing was then performed on the Illumina HiSeq platform. The raw reads were checked and filtered by the following methods: 1) reads with adapters were removed; 2) reads with low-quality and N bases (quality value ≤10) were removed; 3) to gain a clearer understanding of the bacterial genome data, the host genome data were filtered out by eliminating fall webworm genome sequences.

Kraken [193] was used for the taxonomic identification and relative abundance calculations, and the NCBI Reference Sequence Database (RefSeq), which includes high-quality bacterial, archaea and virus data, could further filter the nonbacterial genome sequences. The microbiota composition was visualized by Krona [194] and Python scripts.

De novo assembly was performed by IDBA-UD [195] (parameter: --mink:21, −-maxk:101, −-step:20, −-pre_correction), resulting in sequences greater than 500 bp. The assembly quality was assessed by QUAST [196]. MetaGeneMark [197] was used to perform ab initio gene prediction with the default settings. Prophage prediction was performed by BLAST (E-value: 1E-05) with a local database based on the ACLAME database. Transposable elements, including DNA transposons, long terminal repeats (LTRs), long interspersed elements (LINEs) and short interspersed elements (SINEs), were identified by using RepeatMasker v 4.0.5 and RepeatProteinMasker [161]. A nonredundant data set was outputted by CD-HIT [198] with a minimum coverage cut-off of 0.9 for the shorter sequences. All genes in our nonredundant dataset were translated into amino acid sequences and aligned to relevant databases: NR, COG, KEGG, Swiss-Prot, CAZy and ARDB by BLASTP (E-value ≤1E-05). Blast2GO was used to obtain GO annotations, and HMMER v 3.0 [199] was used to annotate sequences in our dataset from the Pfam database.

### RNA interference with silk fibroin genes

To study the web-producing mechanism in the fall webworm, silk-gland-related genes were identified by analyzing the *H. cunea* genome and the silk gland transcriptome produced in this study. Three genes encoding structural proteins, fibroin heavy (*Fib-H*), fibroin light (*Fib-L*) and protein 25 (*P25*), were silenced by RNA interference (RNAi) to examine their biological functions. RNAi was performed by injecting the corresponding gene-specific double-stranded RNAs (dsRNAs), and green fluorescent protein (*GFP*) was used as a negative control. The dsRNAs for *HcunFib-H*, *HcunFib-L*, *HcunP25* and *GFP* were synthesized by using the MEGAscript RNAi Kit (Ambion, Austin, TX, USA) following the manufacturer’s procedure and purified by lithium chloride precipitation. After quantification with a NanoDrop 2000 (Thermo Fisher Scientific, Wilmington DE, USA) and 1% agarose gel electrophoresis, the dsRNA of the four genes was stored at − 80 °C before use. Then, newly molted third-instar larvae were injected with 4 μg of targeted dsRNA in 1 μL into the abdomen using a Nanoliter 2000 injector (World Precision Instruments, Sarasota, FL, USA). In total, 20 individuals were injected, divided into four plastic boxes (20 cm × 10 cm × 5 cm) and fed fresh mulberry leaves (6 g per box per day); of these, 15 individuals were used to observe the phenotype (*N* = 3), while 5 individuals were used for RT-qPCR validation (*N* = 5). The effect of RNAi was examined by RT-qPCR 4 days after injection; each cDNA sample was quantified based on the total RNA (2 μg) from the 5 insects separately before reverse transcription (SuperScript™ III First-Strand Synthesis SuperMix), and β-actin was employed as an internal control. RT-qPCR was performed on a StepOnePlus Real-Time PCR Detection System (Bio-Rad, Hercules, CA, USA) using TransStar Tip Top Green qPCR Supermix (TransGen Biotech, Beijing, China). The silk web was collected from each box within 10 days after injection. Because the silk filaments were difficult to quantify, they were rolled into a tight ball, and the diameter of the silk ball was used to calculate the silk quantity. The RT-qPCR data were analyzed by the 2^-ΔΔCT^ method. The primers used in this study are listed in Table S14, and the efficiency of each primer pair was tested before the RT-qPCR experiments.

## Supplementary information

**Additional file 1: Figure S1**. K-mer distribution of preprocessed data with k = 19. The distribution of depth analysis based on whole genome data in the fall webworm. Using the formula: genome size = k-mer count/peak of the kmer distribution, thereinto, k = 19.

**Additional file 2: Figure S2.** Numbers of genes annotated with three gene prediction strategies. The final number of genes supported by homologous prediction and transcriptome prediction was 14,688, accounting for a significant proportion (95.88%) of 15,319 (the total number of protein-coding genes), showing the high quality of the prediction.

**Additional file 3: Figure S3.** GO annotation of the *H. cunea* genome. The capital letters on the x-axis indicate the GO categories as listed below, the left y-axis indicates the percentage of genes in each category, and the right y-axis indicates the number of genes in each category.

**Additional file 4: Figure S4.** KOG annotation of the *H. cunea* genome. The capital letters on the x-axis indicate the KOG classification as listed on the right, and the y-axis indicates the number of genes in each classification.

**Additional file 5: Figure S5.** The distribution of Nr homologous genes within the *H. cunea* genome in insect species. The percentage of Nr homologous genes over the *H. cunea* genome were obtained by EVidenceModeler (EVM) with more ten insect species, including *Bombyx mori*, *Danaus plexippus*, *Helicoverpa armigera*, *Papilio xuthus*, *Manduca sexta*, *Spodoptera frugiperda*, *Papilio polytes*, *Tribolium castaneum*, *Spodoptera litura* and *Spodoptera exigua*.

**Additional file 6 Figure S6.** Box plot of FPKM values from different developmental stages and tissues. Box plot of log10 FPKM values aggregated across the 8232 DEGs of the stage RNA sequencing groups and 7733 DEGs of the tissue RNA sequencing groups.

**Additional file 7: Figure S7.** Numbers of alternative splicing events in different tissues and stages of *H. cunea*. The horizontal axis represents the number of alternative splicing events under the corresponding event, and the vertical axis represents the abbreviation of the classification of alternative splicing events. (1) AE: Alternative exon ends; (2) IR: Intron retention (IR_ON, IR_OFF pair); (3) MIR: Multi-IR (MIR_ON, MIR_OFF pair); (4) MSKIP: Multiexon SKIP (MSKIP ON, MSKIP OFF pair); (5) SKIP: Skipped exon (SKIP ON, SKIP OFF pair); (6) TSS: Alternative 5′ first exon (transcription start site); (7) TTS: Alternative 3′ last exon (transcription terminal site); (8) XAE: Approximate AE; (9) XIR: Approximate IR (XIR ON, XIR OFF pair); (10) XMIR: Approximate MIR (XMIR ON, XMIR OFF pair); (11) XMSKIP: Approximate MSKIP (XMSKIP ON, XMSKIP OFF pair); (12) XSKIP: Approximate SKIP (XSKIP ON, XSKIP OFF pair).

**Additional file 8: Figure S8.** COG functional enrichment analysis annotation of the metagenomic data. L, R, C, G and O are the top five gene function categories. The remaining categories were defined as “Others” which including N: cell motility; D: Cell cycle control, cell division, chromosome partitioning; A: RNA processing and moditication; M: Cell wall/membrance/envelope biogenesis; U: Intracellular trafficking,secretion, and vesicular transport.

**Additional file 9: Figure S9.** Proportion of Carbohydrate-Active EnZymes in the metagenome data. BLASTp was used to compare the sequences of the nonredundant gene sets with the CAZy database to obtain the gene annotation information.

**Additional file 10: Table S1.** Results of the preliminary survey. **Table S2.** Prediction of noncoding genes. **Table S3.** Statistical information of two-algebra data by Burrow-Wheeler Aligner (BWA). **Table S4.** Integrity evaluation of Illumina data. **Table S5.** Integrity evaluation of PacBio data by CEGMA. **Table S6.** Integrity evaluation of PacBio data by BUSCO. **Table S7.** DEG statistical results from different stages and tissues. **Table S8.** Sequencing statistics of metagenomic data. **Table S13.** The detail of the genome versions used in this study. **Table S14.** Primers used for RNA interference and RT-qPCR.

**Additional file 11: Table S9.** Annotation results of the metagenome data. **Table S10.** KEGG enrichment analysis of metagenomic genes. **Table S11.** Metagenomic enzymes associated with cellulose and hemicellulose hydrolysis. **Table S12.** Metagenomic gene sets encoding glutathione S-transferase, esterases, and *P450s*.

## Data Availability

The genome data of *H. cunea* have been deposited in the SRA under the accession number SUB5033887.

## Abbreviations

A2M: Alpha-2-macroglobulin
AAs: Auxiliary activities
ABCs: ATP-binding cassette transporters
ASG: Anterior silk gland
CAZy: Carbohydrate-Active enZYmes
CBMs: Carbohydrate binding modules
CCEs: Carboxyl/choline esterases
CEs: Carbohydrate esterases
COG: Cluster of Orthologous Groups
CSPs: Chemosensory Proteins
DEGs: Differential expression genes
dsRNAs: double-stranded RNAs
Fib-H: Fibroin heavy chain gene
Fib-L: Fibroin light chain gene
FMBP-1: Fibroin-modulator-binding protein-1
FPKM: Kilobase of transcript per million fragments mapped
GFP: Green fluorescent protein
GHs: Glycoside hydrolases
GRs: Gustatory Receptors
GSTs: Glutathione S-transferases
GTs: Glycosyltransferases
KEGG: Kyoto Encyclopedia of Genes and Genomes
KOG: euKaryotic Orthologous Groups
KSPIs: Kazal-type serine proteinase inhibitor
LINE: Long Interspersed Elements
LRTs: Likelihood ratio tests
LTR: Long Terminal Repeat
MSG: Middle silk gland
NR: NCBI non-redundant
OBPs: Odorant Binding Proteins
ORs: Odorant Receptors
P25: Protein 25
Pacifastin: Pacifastin-related serine protease inhibitor
PBP: Phosphatidylethanolamine-binding protein
Pfam: Protein family
PLs: Polysaccharide lyases
PSG: Posterior silk gland
RefSeq: Reference Sequence Database
SGFs: Silk gland factors
SINE: Short Interspersed Elements
SPI 2: Silk proteinase inhibitor 2
UGTs: UDP glycosyltransferases
