# Supplementary figures and images for: Genome of the webworm Hyphantria cunea unveils genetic adaptations supporting its rapid invasion and spread
Source: BMC Genomics. 2020 Mar 18;21:242. doi: 10.1186/s12864-020-6629-6 (PMC7079503; doi:10.1186/s12864-020-6629-6)

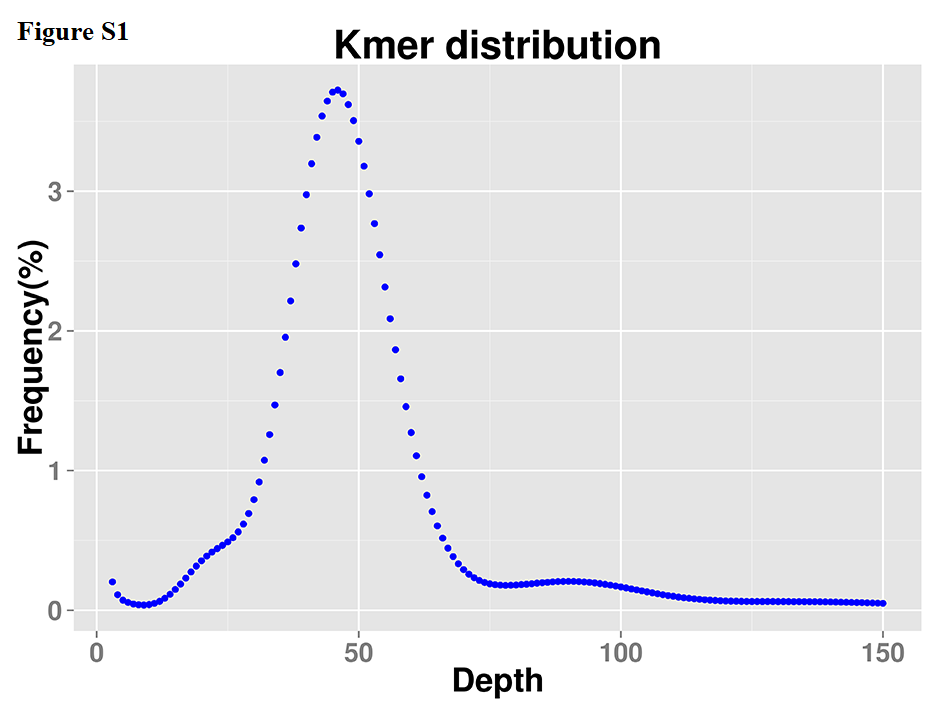

Supplement: Supplementary file 1 — Additional file 1: Figure S1. K-mer distribution of preprocessed data with k = 19. The distribution of depth analysis based on whole genome data in the fall webworm. Using the formula: genome size = k-mer count/peak of the kmer distribution, thereinto, k = 19. [file 12864_2020_6629_MOESM1_ESM.tif]

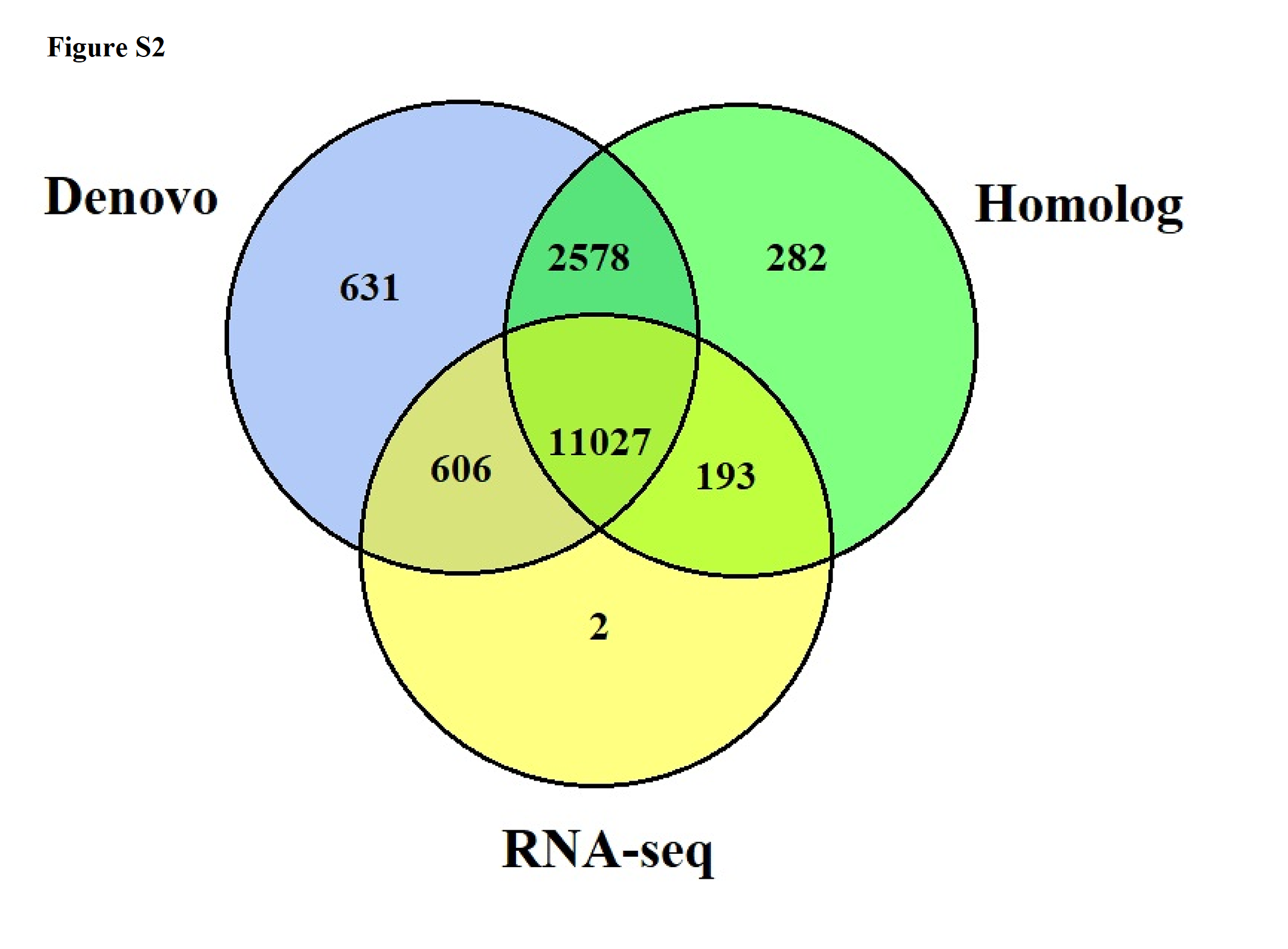

Supplement: Supplementary file 2 — Additional file 2: Figure S2. Numbers of genes annotated with three gene prediction strategies. The final number of genes supported by homologous prediction and transcriptome prediction was 14,688, accounting for a significant proportion (95.88%) of 15,319 (the total number of protein-coding genes), showing the high quality of the prediction. [file 12864_2020_6629_MOESM2_ESM.tif]

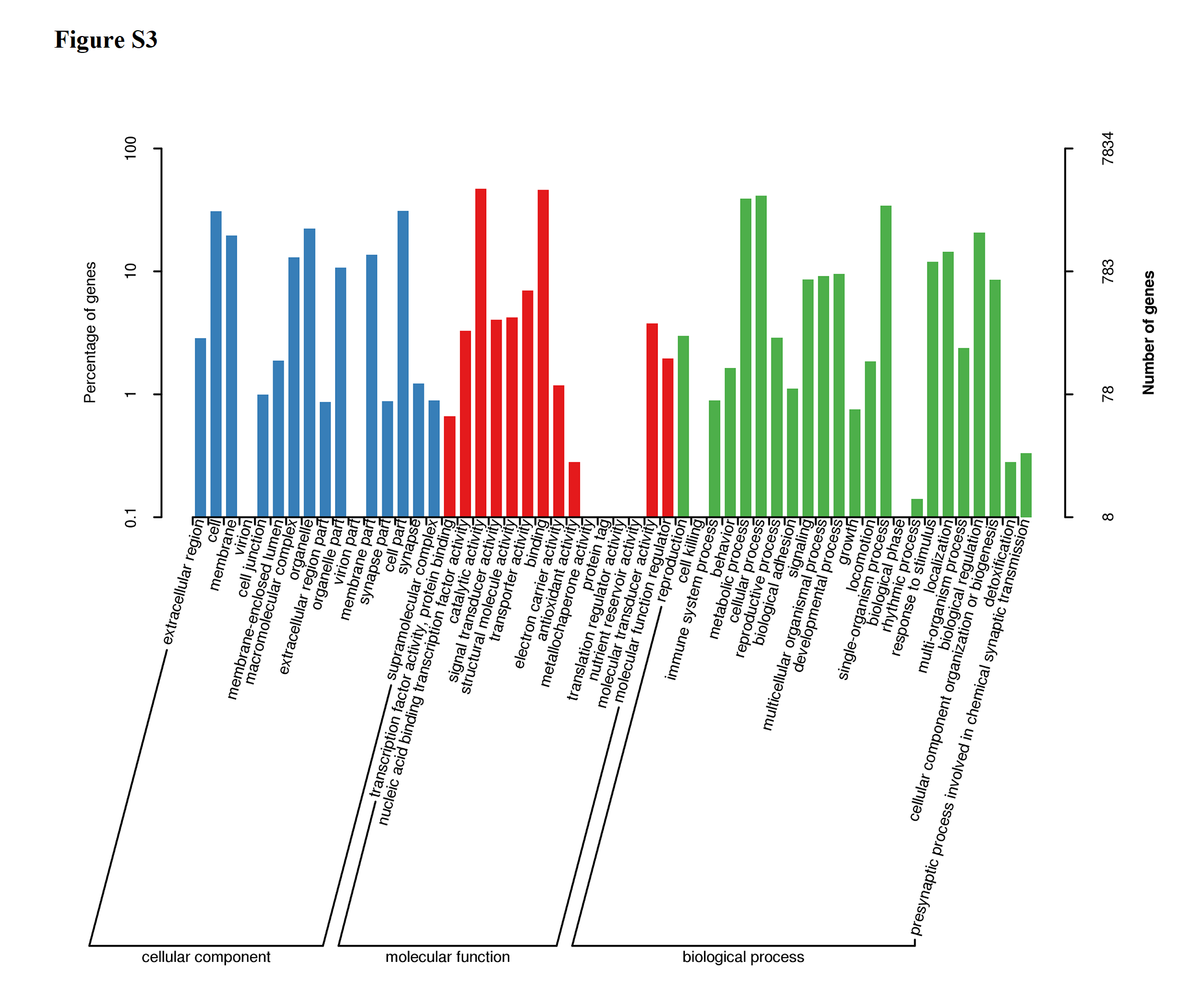

Supplement: Supplementary file 3 — Additional file 3: Figure S3. GO annotation of the H. cunea genome. The capital letters on the x-axis indicate the GO categories as listed below, the left y-axis indicates the percentage of genes in each category, and the right y-axis indicates the number of genes in each category. [file 12864_2020_6629_MOESM3_ESM.tif]

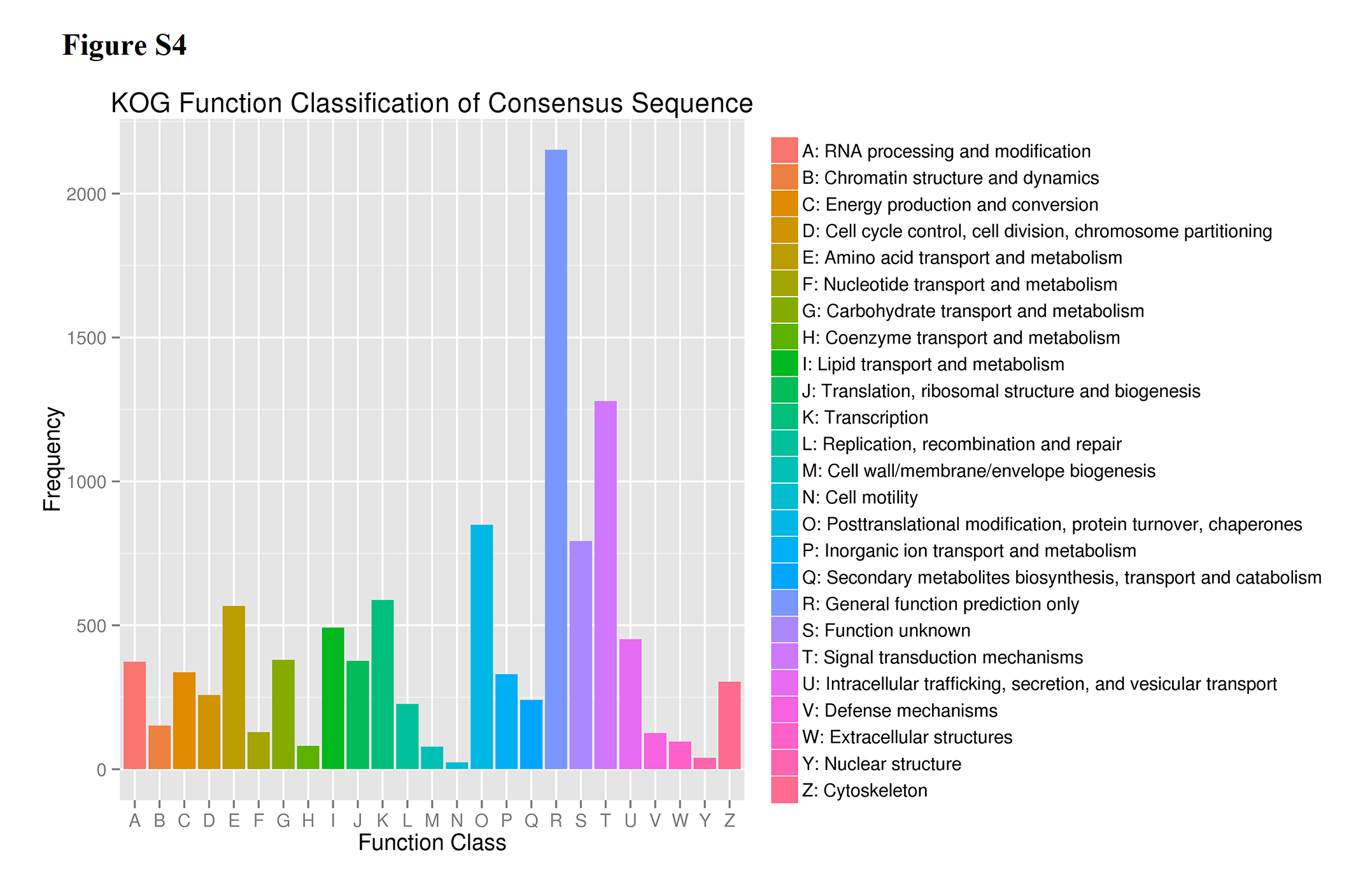

Supplement: Supplementary file 4 — Additional file 4: Figure S4. KOG annotation of the H. cunea genome. The capital letters on the x-axis indicate the KOG classification as listed on the right, and the y-axis indicates the number of genes in each classification. [file 12864_2020_6629_MOESM4_ESM.tif]

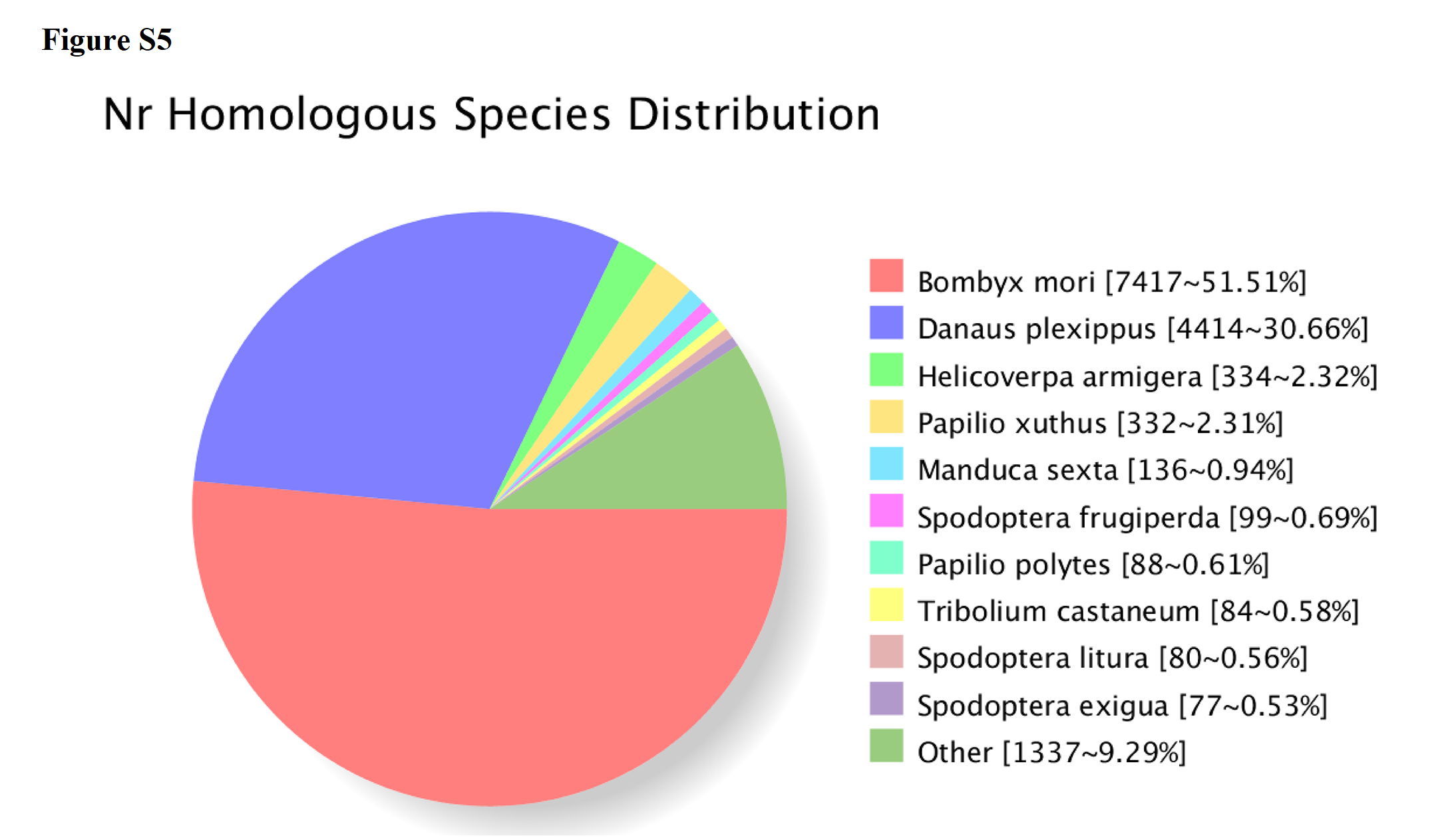

Supplement: Supplementary file 5 — Additional file 5: Figure S5. The distribution of Nr homologous genes within the H. cunea genome in insect species. The percentage of Nr homologous genes over the H. cunea genome were obtained by EVidenceModeler (EVM) with more ten insect species, including Bombyx mori, Danaus plexippus, Helicoverpa armigera, Papilio xuthus, Manduca sexta, Spodoptera frugiperda, Papilio polytes, Tribolium castaneum, Spodoptera litura and Spodoptera exigua. [file 12864_2020_6629_MOESM5_ESM.tif]

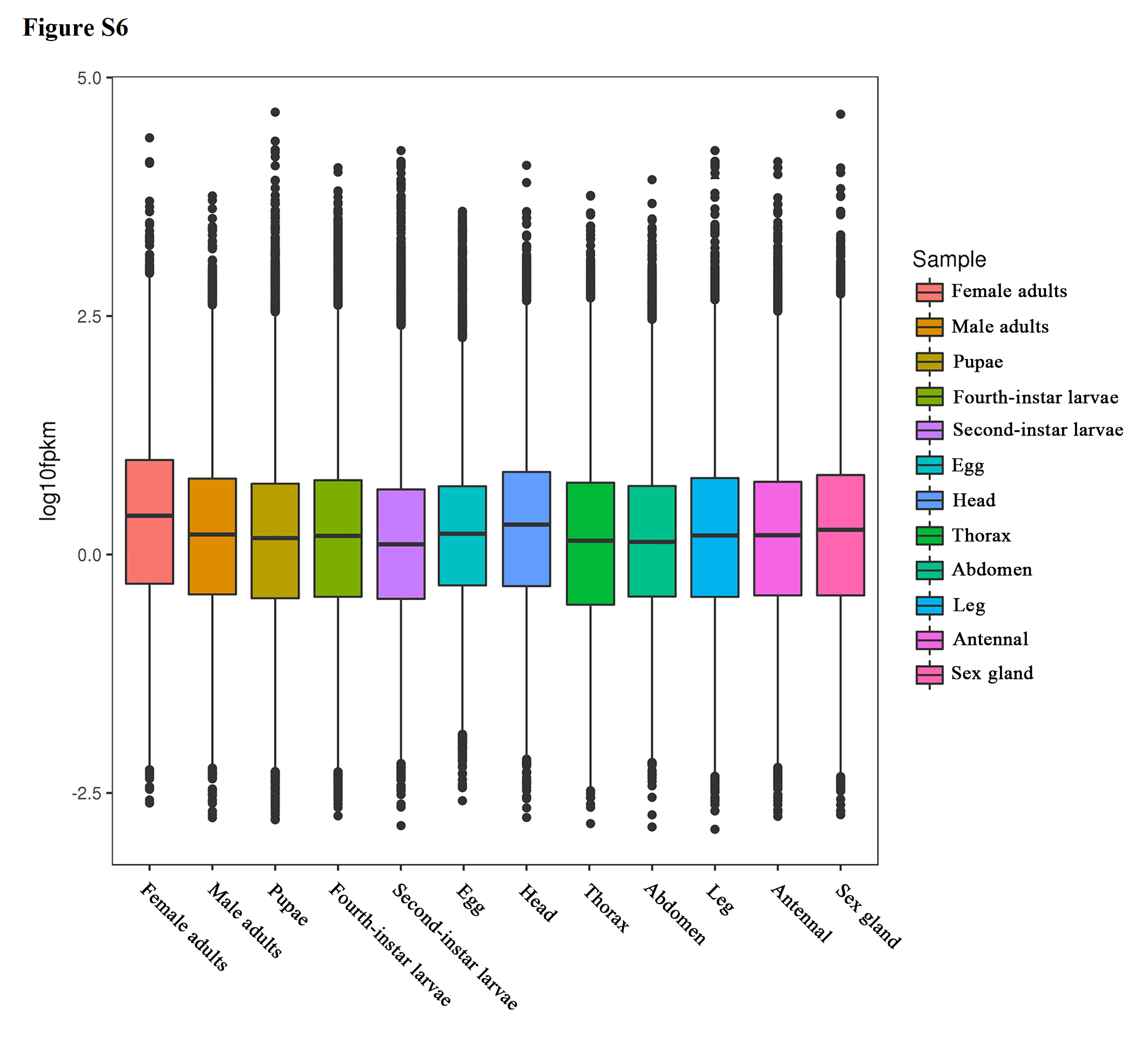

Supplement: Supplementary file 6 — Additional file 6 Figure S6. Box plot of FPKM values from different developmental stages and tissues. Box plot of log10 FPKM values aggregated across the 8232 DEGs of the stage RNA sequencing groups and 7733 DEGs of the tissue RNA sequencing groups. [file 12864_2020_6629_MOESM6_ESM.tif]

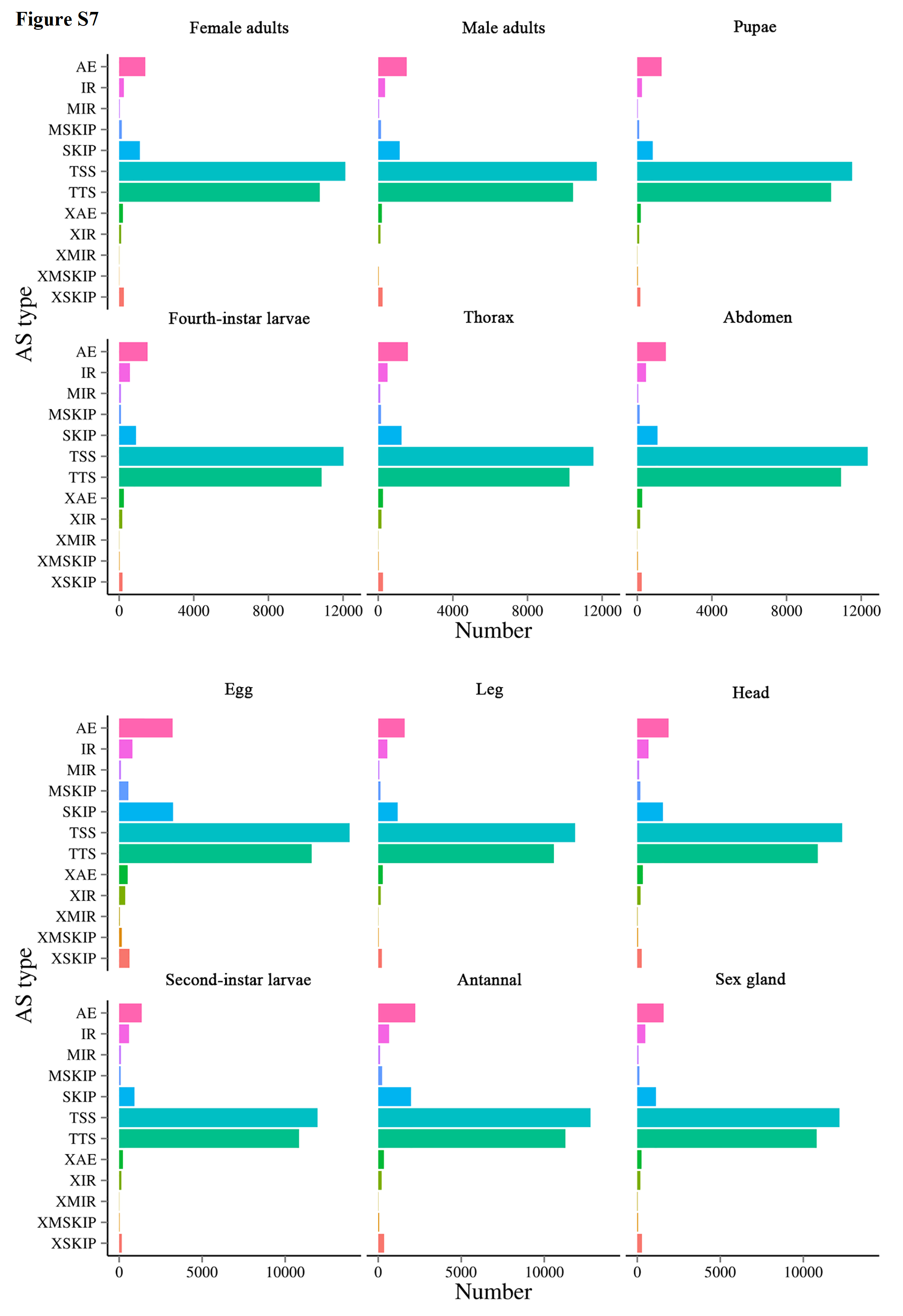

Supplement: Supplementary file 7 — Additional file 7: Figure S7. Numbers of alternative splicing events in different tissues and stages of H. cunea. The horizontal axis represents the number of alternative splicing events under the corresponding event, and the vertical axis represents the abbreviation of the classification of alternative splicing events. (1) AE: Alternative exon ends; (2) IR: Intron retention (IR_ON, IR_OFF pair); (3) MIR: Multi-IR (MIR_ON, MIR_OFF pair); (4) MSKIP: Multiexon SKIP (MSKIP ON, MSKIP OFF pair); (5) SKIP: Skipped exon (SKIP ON, SKIP OFF pair); (6) TSS: Alternative 5′ first exon (transcription start site); (7) TTS: Alternative 3′ last exon (transcription terminal site); (8) XAE: Approximate AE; (9) XIR: Approximate IR (XIR ON, XIR OFF pair); (10) XMIR: Approximate MIR (XMIR ON, XMIR OFF pair); (11) XMSKIP: Approximate MSKIP (XMSKIP ON, XMSKIP OFF pair); (12) XSKIP: Approximate SKIP (XSKIP ON, XSKIP OFF pair). [file 12864_2020_6629_MOESM7_ESM.tif]

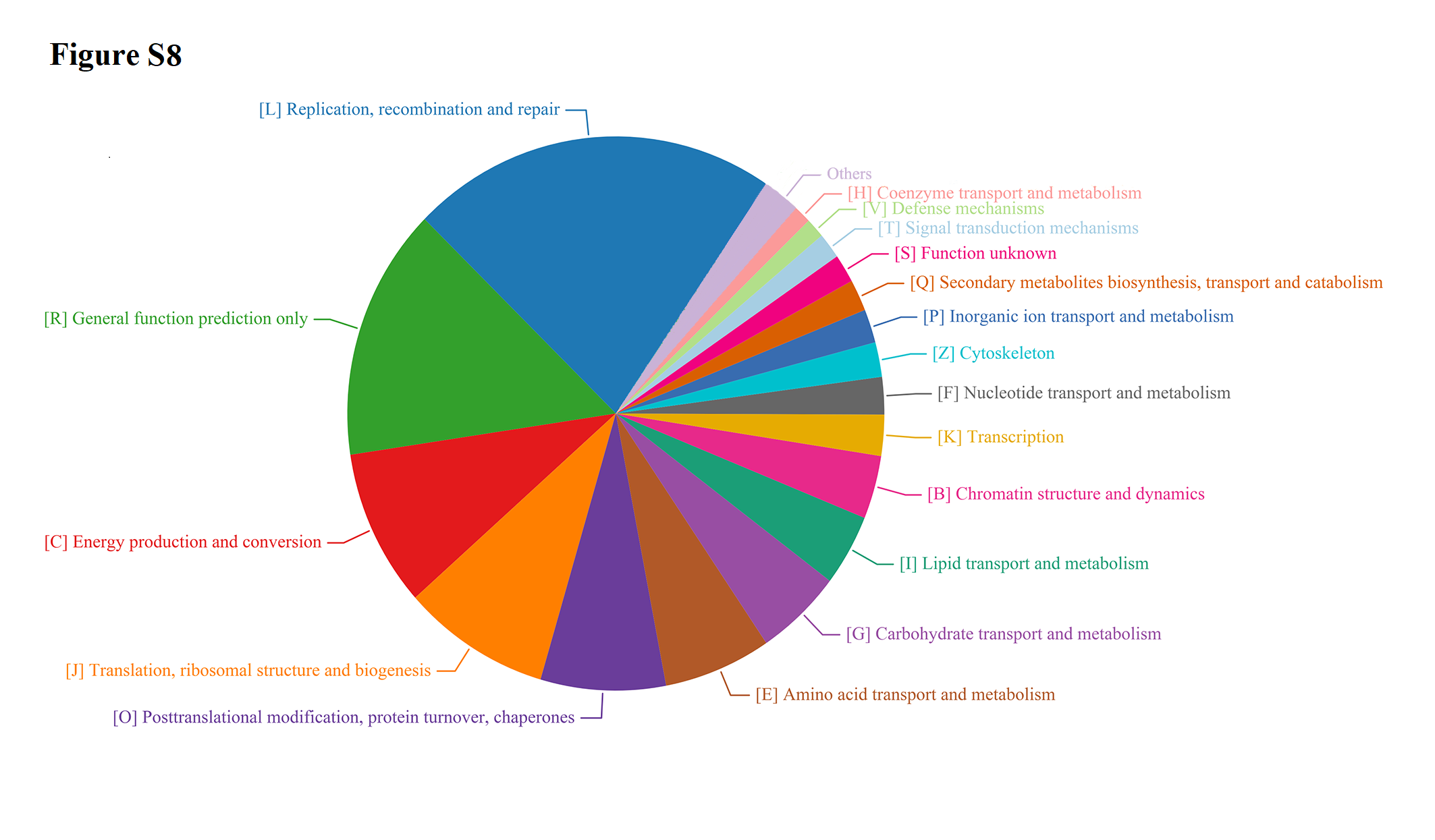

Supplement: Supplementary file 8 — Additional file 8: Figure S8. COG functional enrichment analysis annotation of the metagenomic data. L, R, C, G and O are the top five gene function categories. The remaining categories were defined as “Others” which including N: cell motility; D: Cell cycle control, cell division, chromosome partitioning; A: RNA processing and moditication; M: Cell wall/membrance/envelope biogenesis; U: Intracellular trafficking,secretion, and vesicular transport. [file 12864_2020_6629_MOESM8_ESM.tif]

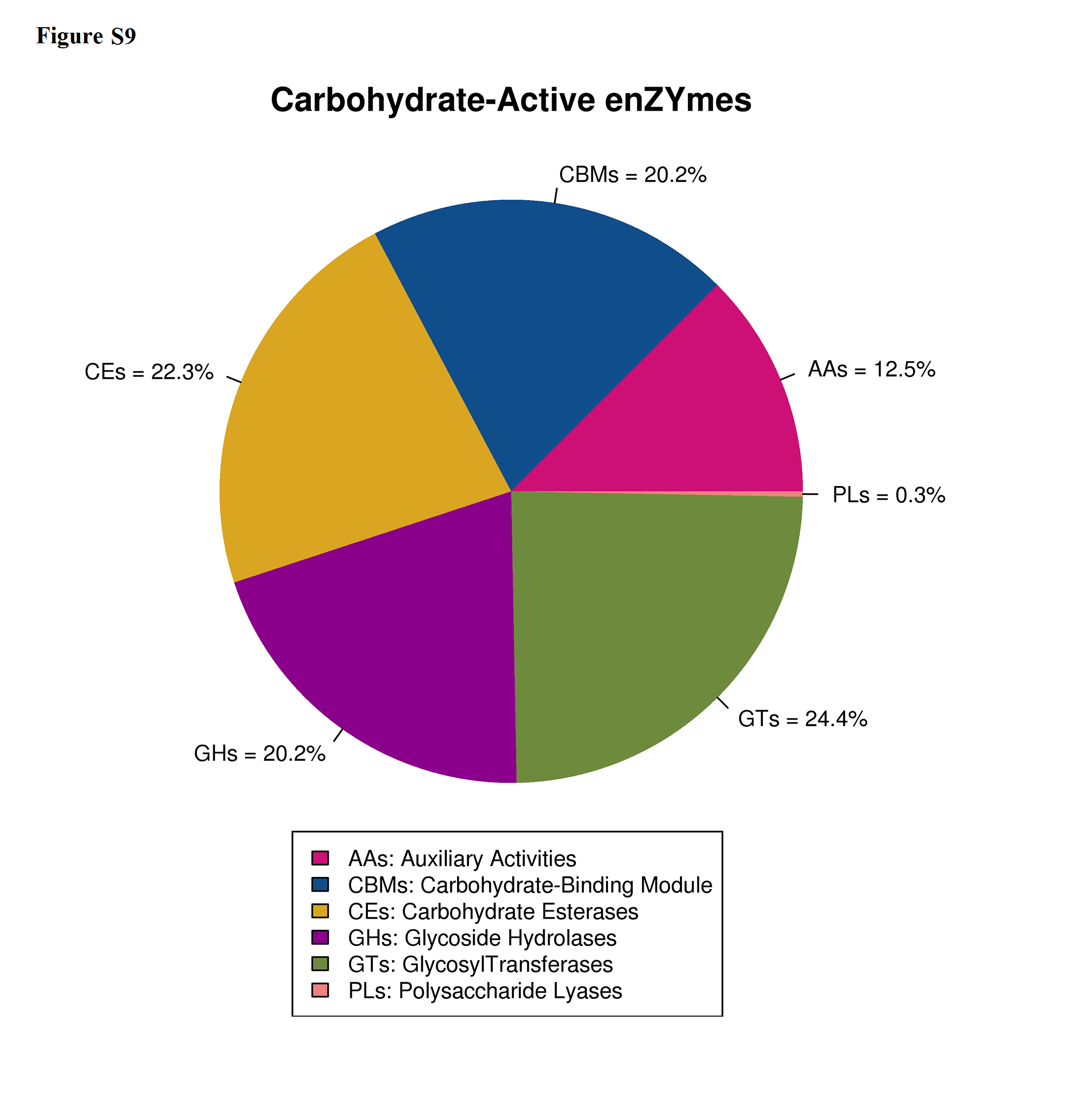

Supplement: Supplementary file 9 — Additional file 9: Figure S9. Proportion of Carbohydrate-Active EnZymes in the metagenome data. BLASTp was used to compare the sequences of the nonredundant gene sets with the CAZy database to obtain the gene annotation information. [file 12864_2020_6629_MOESM9_ESM.tif]
