# Supplementary material for: Genome of the webworm Hyphantria cunea unveils genetic adaptations supporting its rapid invasion and spread
Source: BMC Genomics. 2020 Mar 18;21:242. doi: 10.1186/s12864-020-6629-6 (PMC7079503; doi:10.1186/s12864-020-6629-6)
Supplement: Supplementary file 10 — Additional file 10: Table S1. Results of the preliminary survey. Table S2. Prediction of noncoding genes. Table S3. Statistical information of two-algebra data by Burrow-Wheeler Aligner (BWA). Table S4. Integrity evaluation of Illumina data. Table S5. Integrity evaluation of PacBio data by CEGMA. Table S6. Integrity evaluation of PacBio data by BUSCO. Table S7. DEG statistical results from different stages and tissues. Table S8. Sequencing statistics of metagenomic data. Table S13. The detail of the genome versions used in this study. Table S14. Primers used for RNA interference and RT-qPCR. [file 12864_2020_6629_MOESM10_ESM.doc]

**Table S1. Results of the preliminary survey**.

| Survey genome size | Repeats (%) | GC content (%) | Heterozygosity (%) |
| --- | --- | --- | --- |
| 563.96 Mb | 30.63 | 36.20 | 0.23 |

**Table S2**. Prediction of non-coding genes.

| Classification | Numbers | Family |
| --- | --- | --- |
| miRNA | 48 | 34 |
| rRNA | 71 | 4 |
| tRNA | 637 | 24 |
| Pseudogene | 300 | - |

**Table S3. Statistical information of two-algebra data by burrow-wheeler aligner (BWA).**

| **Library** | **Total reads** | **Mapped reads** | **Mapped (%)** | **Properly mapped reads** | **Properly mapped (%)** |
| --- | --- | --- | --- | --- | --- |
| 270bp | 205671718 | 201702045 | 98.07% | 186165340 | 92.03% |

**Table S4. Integrity evaluation on Illumina data.**

| **Different indexes** | **Data** |
| --- | --- |
| Total number | 2,517,236 |
| Total length | 26,048,932,954 |
| Total map number | 2,474,369 |
| Total map length | 25,596,009,095 |
| Mapping identity | 96.53% |
| Proper map number | 2,353,070(93.48%) |
| Proper map length | 23,877,959,050(91.67%) |
| Proper mapping identity | 96.78% |

**Table S5. Integrity evaluation on PacBio data by CEGMA**.

| **Species** | **Number of 458**  **CEGs* present**  **in assembly** | **% of 458**  **CEGs present**  **in assemblies** | **Number of 248**  **highly conserved**  **CEGs present** | **% of 248 highly**  **conserved CEGs**  **present** |
| --- | --- | --- | --- | --- |
| *Hyphantria cunea* | 433 | 94.54% | 204 | 82.26% |

**Table S6. Integrity evaluation on PacBio data by BUSCO**.

| **Species** | **Complete**  **BUSCOs** | **Complete and**  **single-copy**  **BUSCOs** | **Complete and**  **duplicated**  **BUSCOs** | **Fragmented BUSCOs** | **Missing**  **BUSCOs** |
| --- | --- | --- | --- | --- | --- |
| *Hyphantria cunea* | 991 | 925 | 66 | 15 | 60 |

**Table S7. DEG statistic results from different stages and t**issues.

| **DEG Set** | **DEG Number** | **up-regulated** | **down-regulated** |
| --- | --- | --- | --- |
| Male adults VS Female adults | 2,082 | 672 | 1,410 |
| Female adults VS Pupae | 2,764 | 1,540 | 1,224 |
| Male adults VS Pupae | 2,962 | 1,464 | 1,498 |
| Fourth-instar larvae VS Female adults | 2,962 | 1,403 | 1,559 |
| Fourth-instar larvae VS Male adults | 2,504 | 1,180 | 1,324 |
| Fourth-instar larvae VS Pupae | 2,553 | 1,468 | 1,085 |
| Thorax VS Abdomen | 2,449 | 1,792 | 657 |
| Thorax VS Leg | 2,068 | 1,108 | 960 |
| Thorax VS Head | 2,209 | 1,171 | 1,038 |
| Thorax VS Antennal | 2,616 | 1,471 | 1,145 |
| Thorax VS Sex gland | 2,636 | 1,518 | 1,118 |
| Abdomen VS Leg | 2,636 | 786 | 1,850 |
| Abdomen VS Head | 2,898 | 1,014 | 1,884 |
| Abdomen VS Antennal | 3,463 | 1,511 | 1,952 |
| Abdomen VS Sex gland | 2,645 | 1,127 | 1,518 |
| Egg VS Female adults | 2,550 | 550 | 2,000 |
| Egg VS Male adults | 2,549 | 1,016 | 1,533 |
| Egg VS Pupae | 2,811 | 1,126 | 1,685 |
| Egg VS Fourth-instar larvae | 2,643 | 788 | 1,855 |
| Egg VS Second-instar larvae | 2,798 | 1,314 | 1,484 |
| Leg VS Head | 2,774 | 1,361 | 1,413 |
| Leg VS Antennal | 2,678 | 1,519 | 1,159 |
| Leg VS Sex gland | 3,077 | 1,669 | 1,408 |
| Head VS Antennal | 2,333 | 1,540 | 793 |
| Head VS Sex gland | 3,256 | 1,837 | 1,419 |
| Second-instar larvae VS Female adults | 2,863 | 1,115 | 1,748 |
| Second-instar larvae VS Male adults | 2,683 | 940 | 1,743 |
| Second-instar larvae VS Pupae | 2,651 | 1,012 | 1,639 |
| Second-instar larvae VS Fourth-instar larvae | 2,331 | 763 | 1,568 |
| Antennal VS Sex gland | 3,435 | 1,597 | 1,838 |
| **Different stages** | **8,232** |  |  |
| **Different tissues** | **7,733** |  |  |
| **Total** | **10,348** |  |  |

**Table S8. The sequences statistics of metagenomic data.**

| Sample ID | Contig Num. | Total Len.(bp) | Largest Len.(bp) | N50  (bp) | Mapped  (%) | Properly Mapped (%) |
| --- | --- | --- | --- | --- | --- | --- |
| D01 | 151448 | 520677701 | 106717 | 6830 | 98.78 | 88.44 |

| **Name** | **Accession number** |
| --- | --- |
| *Apis mellifera* | GCF_003254395.2_Amel_HAv3.1 |
| *Bombyx mori* | GCF_000151625.1_ASM15162v1 |
| *Drosophila melanogaster* | GCF_000001215.4_Release_6_plus_ISO1_MT |
| *Helicoverpa armigera* | GCF_002156985.1_Harm_1.0 |
| *Papilio machaon* | GCF_001298355.1_Pap_ma_1.0 |
| *Papilio polytes* | GCF_000836215.1_Ppol_1.0 |
| *Papilio xuthus* | GCF_000836235.1_Pxut_1.0 |
| *Pieris rapae* | GCF_001856805.1_P_rapae_3842_assembly_v2 |
| *Plutella xylostella* | GCF_000330985.1_DBM_FJ_V1.1 |
| *Tribolium castaneum* | GCF_000002335.3_Tcas5.2 |
| *Operophtera brumata* | GCA_001266575.1_ASM126657v1 |

**Table S13. The detail of the genome versions used in this study.**

**Table S14**. The primers used for RNA interference and RT-qPCR.

|  | **Primers for dsRNA Synthesis** | **Primers for RT-qPCR** |
| --- | --- | --- |
| ds*HcunP25*-Sence | **TAATACGACTCACTATAGGGAGA**CTTCCACTACGACACGCCATACTTC | GTTGGGGGCACATTATTG |
| ds*HcunP2*5-Anti | **TAATACGACTCACTATAGGGAGA**CAGATGAGCGAGTCTATGAACTTGTAGG | TATTTGGAGGGCAGGAAG |
| ds*HcunFib-L*-Sence | **TAATACGACTCACTATAGGGAGA**GGGGCACATTATTGCTACG | GTCCTTCCACTACGACACGC |
| ds*HcunFib-L*-Anti | **TAATACGACTCACTATAGGGAGA**TGAAGATGCTCGCTGAACAC | TGACGAAAAAGTCCGAAACAT |
| ds*HcunFib-H*-Sence | **TAATACGACTCACTATAGGGAGA**GTTTCAACTCTCATCACCA | TGGAGATGTTGACGGTACGAGAG |
| ds*HcunFib-H*-Anti | **TAATACGACTCACTATAGGGAGA**CCACGGATACCACCTA | GCTTCGGCTGCTGCTTCTTC |

(T7: TAATACGACTCACTATAGGGAGA was added to the 5’ end of each pair of primers for dsRNA Synthesis.)
